# Life Cycle Assessment of Compostable Coffee Pods: A US University Based Case Study

**DOI:** 10.1038/s41598-020-65058-1

**Published:** 2020-06-08

**Authors:** Komal Kooduvalli, Uday Kumar Vaidya, Soydan Ozcan

**Affiliations:** 1 Fibers and Composites Manufacturing Facility, The University of Tennessee, 1321 White Avenue, Knoxville, TN 37996 USA; 2 Energy and Transportation Science Division, Oak Ridge National Laboratory, 2360 Cherahala Boulevard NTRC- 3, Knoxville, TN 37932 USA; 3 Institute for Advanced Composites Manufacturing Innovation, 2360 Cherahala Boulevard, Knoxville, TN 37932 USA

**Keywords:** Environmental impact, Sustainability, Biomaterials

## Abstract

Single-serve machines have proven to be a rapid and convenient mechanism for preparing coffee for consumption. However, disposing the single-use coffee pods accompanying each use creates insurmountable waste in landfills. With the introduction of biobased products being certified as industrially compostable, there is scope for an effective waste stream for nearly all biobased products that avoids adding to landfills. The case presented in this paper demonstrates the success of composting compostable coffee pods within a local industrial-scale composting facility. Utilizing the existing local composting facility at the University of Tennessee–Knoxville, a life cycle assessment was performed to calculate the overall embodied energy and related environmental impact(s) to determine the feasibility of using compostable coffee pods over conventional plastic ones. Testing showed complete degradation within 46 days, proving composting to be a feasible waste stream option and a sustainable marketing edge while treading the path toward a circular economy. Cost savings of 21% were realized in terms of waste disposal, in addition to creating a value-added product at the end of the coffee pods life cycle, with nutrient-rich compost being recirculated to campus gardens and farms.

## Introduction

Advances in modern technology have allowed for convenient waste disposal options incorporating pathways such as landfilling, incineration, recycling, and composting for applicable materials^1^. However, a rapid rise in population in conjunction with exorbitant resource extraction has caused increasing waste volumes. Currently, landfills and oceans overflow with a myriad of materials, some of which can degrade on human timescales, whereas others may not decompose for hundreds or thousands of years. Furthermore, today’s fast-paced lifestyles do not allow us to fathom the amount of energy, resources, and labor that goes into creating consumer products^2^. With single-cup coffee brewers now placed in ~40% of US workplaces, plastic coffee pods are compounding the issue of recycling complex plastics^3^. Some studies estimate that pods landfilled in 2014 could circle the earth more than 12 times^4,5^.

For reasons relating to convenience, time, and sanitation, many people choose to use disposable coffee pods. While most pods are made from synthetic, noncompostable plastics, companies are now beginning to produce compostable, bioderived plastics. These compostable pods can be sent to industrial composting facilities with the coffee grounds intact, providing convenience for consumers compared to conventional pods while diverting waste from landfills. Coffee pods within our study will be referred to as plastic (i.e., nonbiodegradable) and compostable pods. Each type of pod is described under section *Coffee Pods*.

### Certification

Various organizations assist companies in testing and branding their products as they strive to adhere to standards for a better environmental portfolio^6^. For example, products are deemed sustainably harvested if they are approved for a Forest Stewardship Council (FSC)^7^ or Marine Stewardship Council (MSC) certification^8^. Others consider factors related to recycled content (100% recycled), harmful chemicals (e.g., chlorine-free), and overall systems such as Leadership in Energy and Environmental Design (LEED)^9^ for buildings. In the present study, the most relevant certification has to do with compostable products. Biodegradable Products Institute (BPI)^10^ is the leading organization in the United States for certifying products that meet ASTM D6400 and D6868 standards for industrial composting^11–13^. They consist of a large repository of verified compostable products on the market.

### Sustainability

When assessing the sustainability of a product or process, standardized metrics are required for evaluation^14^. Recycling certain items is more energy intensive than creating them from virgin material considering factors such as age, type of technology, number of recycling cycles, and material composition^15^. For example, although some coffee pods are recyclable, only clean ones can be recycled, and washing would consume energy. By contrast, compostable pods contaminated with coffee grounds would not hinder compostability^16^. Consumers require an accessible yet effective solution that maintains convenience alongside preserving the environment.

### Coffee pods

Single-serve coffee machines have revolutionized the way coffee is prepared and consumed. However, issues surrounding the coffee pods used for these machines have attracted attention in recent years due to the insurmountable waste stream that is produced from discarded conventional pods^17^. Products that can be recycled are placed under seven categories, and are indicated by the universal recycling symbol^18,19^ There are reported instances of pods made from a combination composite plastic material categorized as a #7 plastic (under the “other plastics” category), which cannot be widely recycled. This realization prompted some companies to change to #5 plastics—namely polypropylene (PP)—which kept costs low and recyclability high^20,21^,

Coffee pods can be categorized based on their End-of-Life (EOL) or waste stream options. There are generally three types of coffee pods, as described below.

#### Recyclable pods

Without the ease of accessible receptacles for recycling, most of the pods end up in landfills. It has been reported that only 6–9% of plastics get recycled^22,23^. The fact that newer coffee pods are recyclable does not imply that all will be recycled. In the United States, only 9% of plastics, 34% of metals, and 67% of paper from municipal solid waste were recycled in 2015^24^. In addition, recycling pods would prove difficult as the different materials within the pod would have to be separated in order to be recycled, including removal of coffee grounds^25^. Furthermore, many recycling facilities lack the technology to reliably detect the small components of a pod^26^. Although conventional pods may be recyclable, this does not absolve them from other environmental impacts, which are explored in this paper.

Thermoplastics are capable of being recycled multiple times once cured, due to the material’s ability to be remolded after being shaped into a product. Current production lines produce pods made of PP that have been either thermoformed or injection molded^20,27^. However, thermoset plastics cannot be recycled. When these plastics are cured, the chains in the molecular structure become interlocked, which hinders the ability to reform the material.

#### Compostable pods

Composting differs from landfilling in important ways. Composting facilitates the biodegradation of organic substances to create a usable agricultural product. Therefore, this waste stream excludes substances that are not biodegradable, unlike landfilling. In addition, composting is intended to create a product (i.e., a nutritious material) that can be useful in maintaining soil health for growing crops^28^. Landfilling may or may not produce useful products, as only some landfills have methane-capture mechanisms that can be used to generate electricity. This capability is not mandatory and is not included at all landfilling sites^29^. Types of composting include aerated windrow (i.e., piling organic matter in long rows); in-vessel; aerated static; and onsite. In terms of processes, composting varies in scale, feasibility, cost, and environmental impacts^30^.

**Figure 1:**
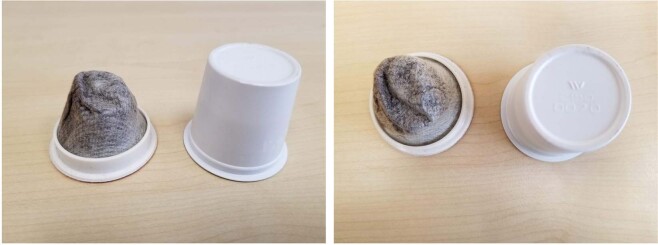
(**a**) Side view of a compostable and plastic coffee pod. (**b**) Top-view of a compostable and plastic coffee pod.

Industrial-scale composting can be utilized at the municipal level, providing convenience and accessibility for the community. This method also provides an efficient way for communities to compost materials collected either at drop-off sites or directly at residences, depending on the area’s consolidation method^31^. Industrial-scale composting allows for a variety of biodegradable materials to be composted due to higher mound temperatures in the larger piles of compostable material, resulting in higher temperatures achieved due to compaction with an organic-rich mound center (approximately 3–6 m high), while diverting waste from reaching landfills. Figure 1 displays the top and side views of a compostable and plastic cup in reference to this study.

#### Reusable pods

**Figure 2:**
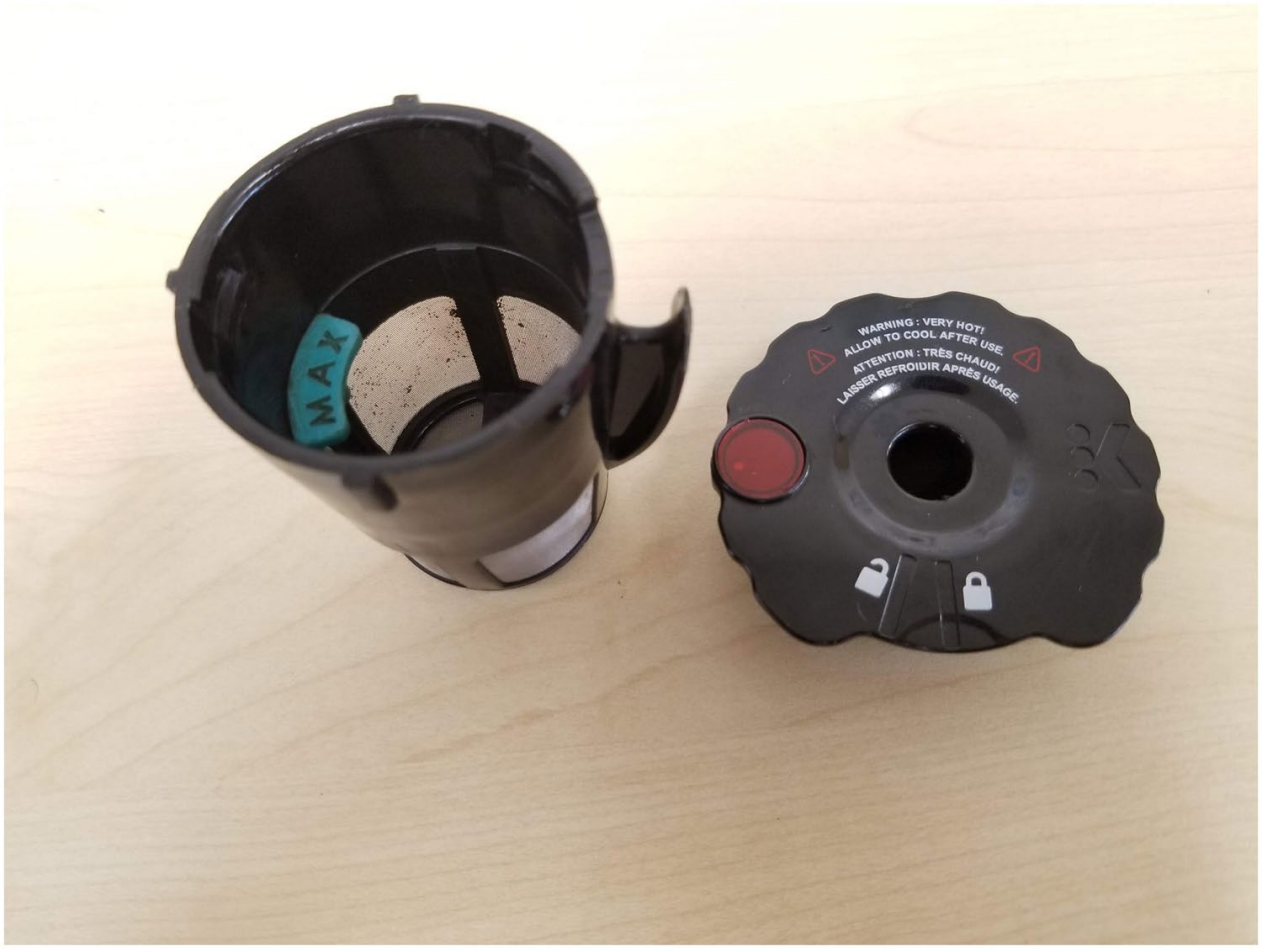
Representative reusable pod.

Reusable coffee pods are ideal for preparing coffee using coffee powder and merely discarding the spent powder after every use (Fig. 2). However, many consumers find the task inconvenient and prefer disposable pods. This is where the advantage of compostable pods is highlighted while maintaining convenience and reducing environmental impacts and overall cost through composting.

#### Landfill scenario

Pods that do not enter either recycling or composting waste stream go to landfills. If the pod is plastic, it remains for hundreds or thousands of years. It is estimated that 9 billion pods ended up in landfills in 2014^32^. With the popularity of disposable coffee pods rising, it can reasonably be assumed that this number will grow unless other waste stream options are made available. In such cases, if the pods were compostable and ended up in landfills, anaerobic conditions would ensue, releasing methane in such environments, which could feed into methane-capture systems for the generation of electricity if such capacity exists, or be emitted into the atmosphere^33^.

#### Landfill versus composting emissions

Aerated composting is sustainable for two reasons. First, aeration allows for a higher carbon dioxide (CO_2_)-to-methane (CH_4_) ratio in terms of greenhouse gas emissions^28^. Owing to methane’s global warming potential (GWP) of 28–36 for 100 years and 84–87 for 20 years compared to 1 for CO_2_ (baseline), reducing methane emissions significantly is important for reducing more immediate impacts on the climate^34^. Second, landfills serve little purpose besides electricity generation through methane-capture systems. Some research is focused on repurposing landfills for pollinators and installing renewable energy systems^35–38^. This still does not prevent additives and plastic compounds from leaching into the environment and does not address the fact that plastic products are deemed unusable due to loss of material recovery in landfills^39^. Compost not only provides nutrient additive to soil, but may reduce chemical fertilizer input in some cases^40^.

## Materials and Methods

**Figure 3:**
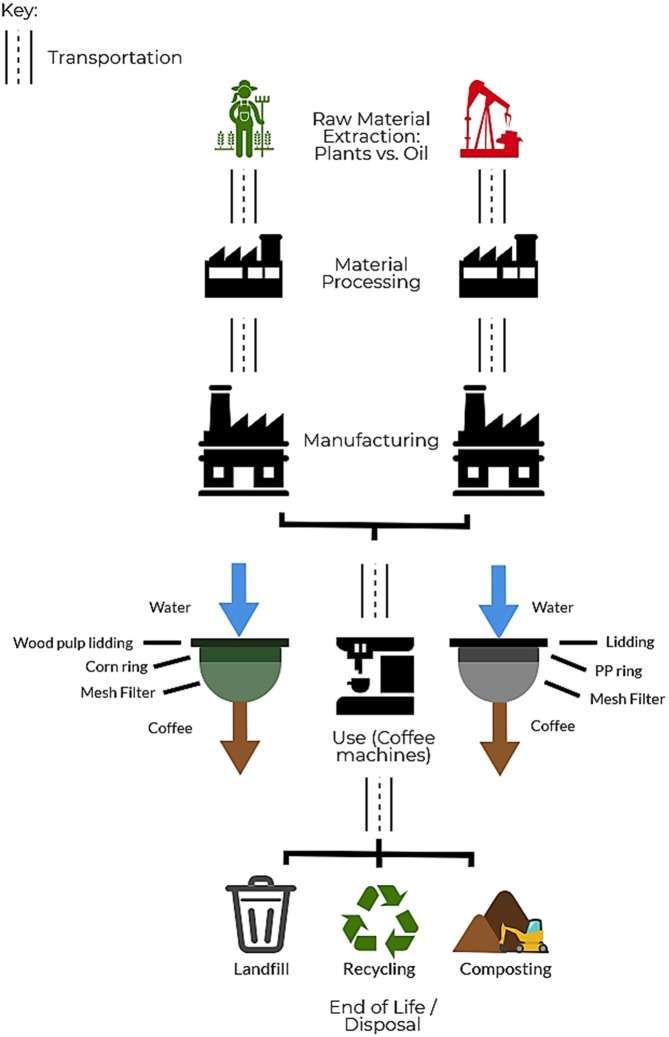
Life cycle schematic of a conventional plastic coffee pod and a compostable coffee pod.

The University of Tennessee (UTK), Knoxville has conducted composting on campus for several years, transitioning from coffee grounds only to include all of campus’ postconsumer dining, yard, and agricultural waste. Following a windrow-style composting setup in which large mounds of material are arranged in lines, the scale of this method of composting allows for proper conversion of biobased products into usable compost. The mounds are turned every other day in 15-day cycles and then allowed to mature for 6 months^41,42^. Figure 3 represents each step of the supply chain for conventional and compostable coffee pods. Details regarding weights and data sources of each of the modeled pod components can be found under section *Data Quality*.

### Timeline and progression

To test the degradation rate of pod samples within the composting environment, the samples were encapsulated within a 30.5 cm × 25.4 cm wire mesh cage and tied to brightly colored nylon ropes for easy identification. Samples were retrieved with the help of the compost site operator using an excavator, which was followed by weighing and analyzing. During the second phase, wherein the components became smaller than the holes of the mesh cage, nylon bags with 2 mm diameter pores were used. This procedure is adapted from the ASTM D5338 and EN13432 standards^43,44^, which note that degradation of a compostable material is considered complete once the particles are small enough to pass through a 2 mm wide sieve and no more than 10 percent of the original amount remains. This experiment ran from June 4 to July 202018,(46 days).

### Pod usage within the university campus

A database (facilities portal) that holds floor plans for every building on campus was used as a basis for this work^45^. In conjunction with the university campus map^46^, points of interest applicable for this study were noted. In this context, single-serve coffee machines were assumed to be centralized in educational labs, offices, classrooms, research facilities, and residential halls. Paired with the following assumptions, these points of interest provided a low estimate of coffee pods’ minimum impact.

*Condition 1*: Consumers use single-serve coffee machines in areas of personal study, rest, and congregation. Areas satisfying this condition include office spaces, self-serve dining areas, meeting rooms, and dormitories.

*Condition 2*: Any floor containing at least one of the spaces mentioned in condition 1 is assumed to contain a single-serve coffee machine. If a floor is assumed to have more than one single-serve coffee machine based on capacity, the next value is considered irrelevant to prevent overestimation.

**Figure 4:**
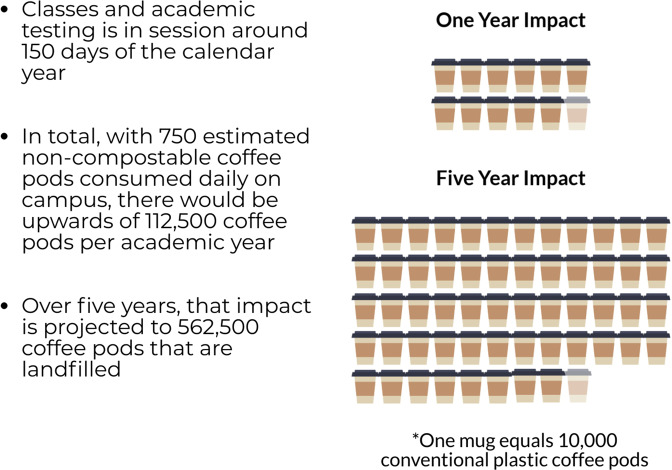
Annual and 5-year pod consumption projections at the university level.

The appendix provides a detailed explanation of the source of coffee pods and assumptions used in this study. It was assumed that at least 750 single-serve coffee pods are consumed daily on campus (value rounded up from 748). It was also assumed that one person will drink one pod per day for each coffee machine. Therefore, 750 coffee pods is the conservative estimate of each day’s consumption. Projections for pod usage according to the number of academic days in session (150 days) for annual and 5-year timelines are 112,500 and 562,500 cups, respectively (Fig. 4).

### Life cycle assessment

In the present study, a complete life cycle assessment (LCA) was performed, which is a comprehensive assessment of a product, process, or service. The method accounts for a cradle-to-gate boundary with transport and EOL options included. Associated environmental impacts of procuring and disposing of compostable coffee pod packaging were accounted for in the study. Compostable pods were compared to the conventional plastic pods typically used on the university campus.

#### Goal, scope, and functional unit

The goal of the present study was to perform a cradle-to-gate comparative LCA, including transportation to the customer and EOL phases. This study compared the packaging elements of a plastic single-serve coffee pod with that of a compostable single-serve pod. The functional unit is pod packaging that serves an 8 oz. (236.6 ml) serving of coffee, projected to UTK’s annual consumption considering a university setting. In this comparative study, both plastic and compostable pods were assumed to stay intact without replacement until completion of their single use in a coffee machine. The objective was to quantify the environmental impacts of manufacturing each pod using the Cumulative Energy Demand (CED)^47–49^ and the US Environmental Protection Agency’s (EPA’s) Tool for the Reduction and Assessment of Chemical and other Environmental Impacts (TRACI) methods^50^, with annual projections for transport and EOL sensitivities.

#### Life cycle inventory

The primary data was calculated based on empirical measurements, literature review, and published retail pricing. This holds true for the mass of components, materials, cost per pod, and wet weights. Values for modeling impacts of transportation and waste streams were taken from software databases within SimaPro v.9.0.0.33^51–53^. Details of each process is provided under section Description of Product Systems. All LCA calculations comply with ISO 14040 and 14044 standards.

#### Assumptions and estimations

The following assumptions are considered for manufacturing, transportation, and EOL.

*Manufacturing*

1. Injection-molded polypropylene (PP) coffee pods were used in this study, as evidence suggests that companies have increased their use of PP due to its higher rate of recyclability. Other materials may include polystyrene, polyethylene (PE), ethylene vinyl alcohol copolymer, and some of these use thermoforming. This study is limited to injection-molded PP pods.
2. Both circular lids are assumed to be produced by sheet rolling, calendaring, and extrusion.
3. To examine materials processing, local US average datasets (including electricity) for manufacturing and transport were taken.
  *Transportation*
4. Procurement for certain materials is unknown. Therefore, global market data has been used for modeling most raw materials across both supply chains to consider “product specific transport distance estimations” within the SimaPro Market data^54^. When the supplier for materials is not known, it is advised as a rule of LCA modeling to utilize Market data, which has been applied for the production of both pods.
5. Because final products are assumed to be transported to the university, these distances have been considered within the shipping step.
  *End-of-Life*
6. The original waste type was changed for the polylactic acid (PLA) dataset within the SimaPro US-EI dataset from ‘biopolymer’ to ‘compost’ for the sake of modeling (i.e., renamed to ‘CompostCopy–Polylactide, granulate, at plant/GLO US-EI U’). The same was applied to unbleached Kraft paper (i.e., renamed to ‘CompostCopy -Kraft paper, unbleached, at plant/US- US-EI U’). For the compost scenario, the ‘Biowaste [RoW]’ process was copied, and transportation values coinciding with the current model were added to make them specific to the scenarios considered. Similarly, landfill models for both were altered: _Municipal solid waste [RoW]| treatment of, sanitary landfill | Cut-off, U to include transportation relevant to the university case.
7. Transport of finished products and EOL disposal occurs through trucks and municipal waste collection trucks for municipal waste transport to both landfill and compost facilities.

#### Cut-off criteria

This section indicates specific points of inclusion and exclusion in terms of modeling parameters and cut-off criteria in terms of system boundaries. The mass of each component has been accounted for in terms of literature values or empirical data. The energy for manufacturing each component was derived from default SimaPro manufacturing process databases (i.e., injection molding, thermoforming, calendaring, sheet rolling, extrusion). Table 1 specifies materials, processes, and other modeling consideration for the given study.

**Table 1:** Exclusion and inclusion table.

Exclusion	Inclusion
1. The use phase of coffee machines, amount of nitrogen injected into pods, and the coffee itself (i.e., coffee bean production and roasting) are excluded for both kinds of pods.	1. All materials; components (e.g., lid, mesh, filter, shell, ring); and processes involved in each step of manufacturing both plastic and compostable coffee pods (i.e., injection molding) from cradle to gate with transport to the customer and EOL phases.
2. Although die casting is a part of lid production, due to lack of information, it was left out of the referenced thesis^16^ and in this evaluation.	2. Sensitivity analyses to understand the impact of variation in transportation and relevant EOL waste stream options on the overall model.
3. Compostable lid uses unbleached Kraft paper as a proxy for abaca fiber generally used for coffee filters. Similarly, for plastic cups, bleached Kraft paper is used as a proxy for abaca filter.	3. Projected scenarios are included in Supplementary Tables S11–S14 to inform readers regarding wet weight impacts.
4. Because organic printing ink was not available in any of the databases, this has been left out to avoid error.	4. Manufacturing and packing centers for both pods are assumed to be within the same facility.
5. Although the rim of the filter paper is coated with PE for adhesion to the ring, this process is not specified anywhere in literature and hence was not considered.	
6. Coffee chaff, which the reference thesis^16^ assumed as part of the compostable ring, is excluded here because Biodegradable Products Institute certification for the compostable pod considered in this study specifies only PLA.	
7. Only pods are considered for evaluation, without considering secondary packaging such as cardboard boxes and PLA bags covering the compostable pods.	
8. Material losses were not considered in this model due to percentage uncertainty for the processes specified here.	
9. The use phase of coffee machines, coffee bean production, roasting, and details related to single-serve machines are excluded as the goal is to understand the impacts of packaging.	

#### Data quality

Precision, completeness, consistency, and representativeness are factors used to evaluate data quality for this study comparing the overall environmental impacts of a plastic coffee pod to that of a compostable one.

Precision: Because most values are taken from previously published work and industry data, in addition to experimental weighing, this information can be considered precise and accurate. Primary data was obtained through experimental weighing of individual pod components. Details of dry and wet weights can be found in Table 2. All secondary data was obtained from SimaPro software databases relating to materials and processes, utilizing Ecoinvent 3.4 data (specifically the allocation, cut-off by classification – unit)^55^ and US-EI 2.2^56^ libraries, which are reproducible. These datasets were chosen with the intention of selecting information relevant to those utilized within the United States and its industries. Transformational data from Ecoinvent—information without the inclusion of global average transportation distances—was chosen for those steps wherein transportation distances were known and input manually whereas global data (GLO) was selected for those materials and processes where procured distances were unknown and required transportation estimates (i.e., material used to make both plastic and compostable pods).

**Table 2:** Measured dry and wet weights of coffee pods. *These are the wet weights referenced within the projected scenario results. Wet weights reflect the model closest to the one that facilities services have to consider in terms of disposal.

Product	Empty dry weight of coffee pod	Dry weight	Wet weight*	Difference (water content)
Plastic coffee pod #1	3.0213 g Difference between empty cup to dry weight => Amount of coffee = 8.9787 g = 9.9787 g = 4.9787 g Average coffee weight: = 23.9361/3 = 7.97 g => 8 g	12 g	21 g	9 g
Plastic coffee pod #2		13 g	19 g	6 g
Plastic coffee pod #3		8 g	18 g	10 g
		Average dry weight of plastic pods: 11 g	Average wet weight of plastic pods: 19.3 g	Average water weight: 8 g
Compostable coffee pod	3.0622 g Difference between empty cup to dry weight => Amount of coffee = 11.9378 g = 10.9378 g = 10.9378 g Average coffee weight = 33.8134/2 = 11.27 g => 11 g	15 g 14 g 14 g	24 g 3 g 24 g	9 g 9 g 9g
		Average dry weight of compostable pods: 14.3 g	Average wet weight of compostable pods: 23.6 g	Average water weight: 9 g

Calculated transportation distances, estimations in terms of coffee pod usage, and assumptions are listed in the following sections.

Completeness: No gaps in data have been identified. Those processes not found in the literature had suitable proxies (e.g., Kraft paper as a proxy for abaca fiber). The model is therefore considered complete.

Consistency: All data points were collected, replicating the same methodology and approach.

Representativeness: Representativeness, in terms of data quality, is explained in Tables 2 and 3.

**Table 3:** Weight of each pod component with data source. *Weights and percentages used in the SimaPro model (data from Ecoinvent 3.4 [allocation, cut-off by classification–unit] and US-EI 2.2 libraries)^53^.

Product (1p)	Component*	Type	Dataset with database source	Value	Unit
Plastic coffee pod Total: 3.0213 g (3.0223 g by scale)	Shell total: 1.7908 g + 0.7841 g (ring) = 2.5749 g (85.22%)	Materials	Polypropylene (GLO) Polypropylene, granulate {GLO}| market for | Cut-off, U	2.5749	g
		Processes	Injection molding (US) Injection moulding/US- US-EI U	2.5749	g
	Lid total: 0.2462 g (8.15%)	Materials	Aluminum (24%) (GLO) Aluminium, primary, cast alloy slab from continuous casting {GLO}| market for | Cut-off, U	0.0591	g
			PET coating (12%) (GLO) Polyethylene terephthalate, granulate, amorphous {GLO}| market for | Cut-off, U	0.0295	g
			LDPE (50%) (GLO) Polyethylene, low density, granulate {GLO}| market for | Cut-off, U	0.1231	g
			Printing ink (14%) (GLO) Printing ink, offset, without solvent, in 47.5% solution state {GLO}| market for | Cut-off, U	0.0345	g
		Processes	Sheet rolling (only for Al) (US) Sheet rolling, aluminium/US- US-EI U	0.0591	g
			Calendaring (sheet coating process -PET & LDPE) US Calendering, rigid sheets/US- US-EI U	0.1526	g
			Extrusion (sheet coating process -PET) (US) Extrusion, plastic film/US- US-EI U	0.1526	g
	Filter Total: 0.2002 g (6.63%)	Materials	Kraft paper, bleached (79%) (GLO) Kraft paper, bleached {GLO}| market for | Cut-off, U	0.1582	g
			LDPE (21%) (GLO) Polyethylene, low density, granulate {GLO}| market for | Cut-off, U	0.042	g
		Processes	Extrusion, plastic film (for LDPE) (US) Extrusion, plastic film/US- US-EI U	0.042	g
	Transportation mi to km: 1.60934		Company X 9.1 mi (14.645 km) (US) Transport, lorry 7.5-16t, EURO5/US- US-EI U + dry weight of coffee pod	= 14.645 × 3.0213 = 44.25	gkm
			Landfill (Meadow Branch Landfill, Athens TN) -59.4 mi (95.595 km) Transport, municipal waste collection, lorry 21t/US* US-EI U For the wet weight scenario, water weight of 8 g was added Tap water, at user/US- US-EI U	= 95.595 × 3.0213 = 288.82 = 95.595 × (3.0213+8) = 95.595 × 11.0213 = 1053.58	gkm
Compostable coffee pod Total: 3.0622 g (3.0619 g by scale)	Lid Total: 0.2504 g (8.18%)	Materials	CompostCopy -Polylactide, granulate, at plant/GLO US-EI U (90%)	= 0.22536	g
			Kraft paper, unbleached, at plant/US- US-EI U (10%)	= 0.02504	g
		Processes (Only for PLA)	Extrusion, plastic film/US- US-EI U	= 0.22536	g
			Calendering, rigid sheets/US- US-EI U	= 0.22536	g
	Ring Total: 2.6828 g (87.61%)	Materials	CompostCopy -Polylactide, granulate, at plant/GLO US-EI U	2.6828	g
		Processes	Injection moulding/US- US-EI U	2.6828	g
	Mesh Total: 0.1290 g (4.21%)	Materials	CompostCopy -Polylactide, granulate, at plant/GLO US-EI U	0.1290	g
		Processes	Extrusion, plastic film/US- US-EI U	0.1290	g
	Transportation mi to km: 1.60934		Company A Lincoln, CA -2439 mi (3925.18 km) (US) Transport, lorry 7.5-16t, EURO5/US- US-EI U	= 3925.18 × 3.0622 = 12019.68	gkm
			Landfill (Meadow Branch Landfill, Athens TN) -59.4 mi (95.595 km) Transport, municipal waste collection, lorry 21t/US* US-EI U For the wet weight scenario, water weight of 11 g was added Tap water, at user/US- US-EI U	= 95.595 × 3.0622 = 292.73 = 95.595 × (3.0622+11) = 95.595 × 14.06 = 1344.07	gkm
			Compost Cherokee Trail, Knoxville, TN 37920 -3.9 mi (6.276 km)	= 6.276 × 3.0622 = 19.21	gkm

Geographical representativeness: Geographical boundary caters to US averages and is applied for all manufacturing and transport consideration within the context of the university campus. The TRACI methodology was also employed for this reason. The quality of individual datasets may vary according to age, representativeness, measured values, and estimates.

Temporal scope: The temporal scope for this study is the low-estimate annual consumption of coffee pods within the university campus.

#### Allocation procedure

This study caters to the physical allocation of mass approach by focusing purely on the impact of conventional and compostable coffee pods.

#### Description of product systems

The following tables present empirical and qualitative data obtained for the study. Table 2 gives measurements related to both types of coffee pods, and Table 3 gives data sources with mass allocation for each.

## Results and Discussion

The cost of shipping from the site of disposal to the waste processing facility is not considered in the prices summarized in Table 4. Depending on the availability and proximity of either local waste stream, individual and/or organizational prices may vary. In addition, disposal fees may vary in other areas of the United States. This compares waste stream costs available within the greater Knoxville, Tennessee area. The pod pricing is also subject to change depending on brands and distances. It must be noted that LCIA results are relative expressions and do not predict impacts on category endpoints, the exceeding of thresholds, safety margins or risks.

**Table 4:** Cost per coffee pod, disposal and net total.

Pod type	Cost per pod		Net cost per pod	Costs for 1 year (112,500 pods)		Net total for 1 year	Net total for 5 years (562,500 pods)		Net total for 5 years
1. Compostable coffee pod, lowest price	Pod: $0.35	Disposal: $0.001567^*^^79^	$0.35	Pods: $39,375	Disposal: $176.29	$39551.29	Pods: $197,875	Disposal: $881.44	$197,756.44
2. Compostable coffee pod, closest distance	$0.82	$0.001567^*^ ^79^	$0.82	Pods: $92,250	Disposal: $176.29	$92,426.29	Pods: $461,250	Disposal: $881.44	$462,131.44
3. Compostable coffee pod, optimized distance and price	$0.49	$0.001567*^79^	$0.49	Pods: $55,125	Disposal: $176.29	$55,301.29	Pods: $275,625	Disposal: $881.44	$276,506.44
4. Conventional coffee pod	$0.62	$0.0009341*^80^	$0.62	Pods: $69,750	Disposal: $105.08	$69,855.08	Pods: $348,750	Disposal: $525.38	$349,275.38

^*^Disposal cost values are approximations made by employees working within the university facilities department; therefore, actual values may differ. All values were obtained between mid-2018 through 2019, but this information is subject to variation from one year to another. The cost per pod for each type of coffee pod was obtained from retail prices mentioned for each pod brand.

Compostable coffee pods observed in the experiment, shown in row 1 of Table 4, cost approximately 44% less annually when purchased and composted compared to purchased conventional pods when sent to the landfill, as shown in row 4. Compostable pod distributors that are closer to UTK than other distributors found (row 2) are slightly more expensive than conventional pods, costing about 32% more when including the EOL scenarios. However, another compostable coffee pod distributor with a slightly increased but similar distance to UTK (row 3) costs about 21% less ($0.49 versus $0.62) than the conventional coffee pod including EOL scenarios. In addition, both EOL disposal options cost less on a per-pod basis when compared to the price of purchasing either type of coffee pod. Optimized cost is more heavily influenced by the cost of the pods themselves rather than the EOL waste stream, with composting and/or landfilling comprising less than 1% of the total cost in all four cases considered. However, in view of the overall cost, compostable coffee pods prove to be the most cost-effective option, even with a slightly more expensive waste stream due to composting.

**Figure 5:**
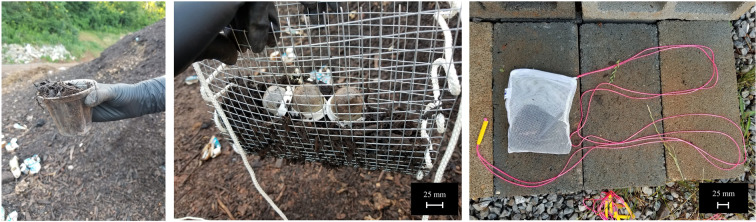
(**a**) Displaying a cup of compost taken from the site. (**b**) Placing the compostable coffee pods into the specimen containers composed of galvanized steel 0.635 cm (0.25 in.) mesh cut into 30.5 cm × 25.4 cm (12 in. × 10 in.) size encapsulation tied with a bright nylon rope, allowing material to degrade with adequate aeration (June 4, 2018). (**c**) Phase 2 encapsulation with nylon bags with pore size of 2 mm with a bright nylon rope tied for easy identification. Each bag was 15.24 cm × 20.32 cm (6 in. × 8 in.) (June 26, 2018).

**Figure 6:**
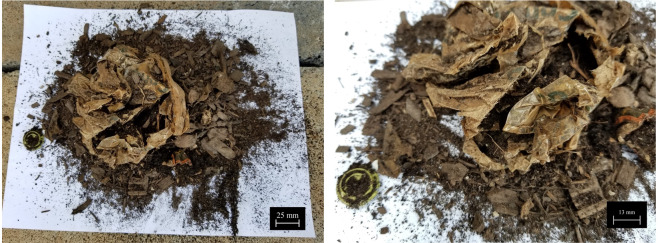
(**a**) Compostable coffee pods and encapsulant PLA bag found within the food mound on July 12, 2018 (38 days within the compost mound). The PLA bag is visible and barely degraded whereas the pods cannot be detected translating to almost complete degradation. This shows that the pods, although structurally sound outside of composting conditions, compost well within conducive conditions within a short timeframe, adding to its environmental value by forming healthy nutrient-rich topsoil. (**b**) Remnants of the compostable PLA bag that formed the encapsulant material for 10 such pods are also seen. The results of the degradation of this bag is outside the scope of this study.

Figure 5 shows the encapsulates for both phases. To seal the cages and identify the specimens, white nylon rope was woven through the gaps in the mesh along the sides, and long strands were left to dangle off of the cage to differentiate it from the compost material. This allowed specimen containment with proper exposure to the industrial composting environment. On July 12, many of the pod specimens had almost completely degraded (Fig. 6).

**Figure 7:**
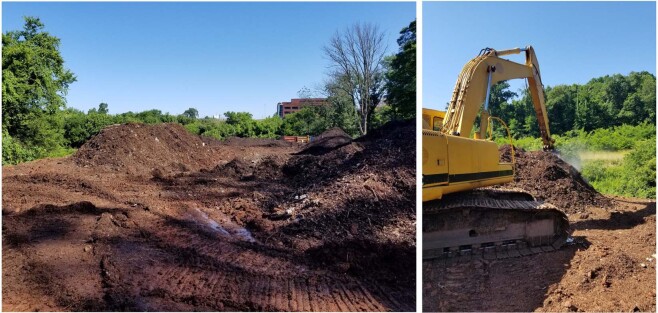
(**a**) View of the compost mounds at the University of Tennessee–Knoxville. (**b**) An excavator is used to periodically turn the compost. The steam can be seen rising from the lifted compost pile as a result of very high temperatures that can reach 100 °C at times, generated from the organic content from within.

Due to the large amount of material that must be aerated in these piles, excavators are required to turn the compost material to allow for even air exposure to all parts of the mound (Fig. 7). This process is carried out at more frequent intervals at the beginning of a turning cycle (approximately every 1–3 days) during the thermophilic period. During this stage, thermophilic bacteria break down substances that are more complex^57^. Once this stage has stopped or slows, the mound is turned less, from weekly to eventually monthly turns, as the mound is allowed to mature. Once a mound has reached maturation, it can be used in various soils.

**Figure 8:**
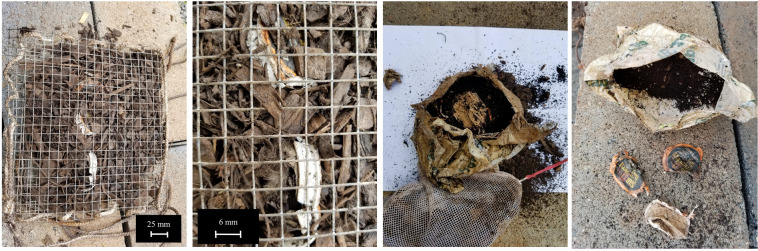
(**a**) Compostable coffee pods with compost material encapsulated within the wire mesh, placed in the mounds on June 4, 2018. (**b**) Close-up of compostable coffee pods having undergone partial composting and stress under pressure and temperature. (**c**) PLA bag taken out during the second phase, where materials were encapsulated in a nylon bag. (**d**) During the final phase, only the paper lids of the pod and the PLA bag remain. Degradation was recorded complete as of July 12, 2018.

The compostable pods were untraceable by the end of the experiment. This shows that the PLA portions can be degraded during a short timescale, by the end of maturation of the mound. In addition, shredding the specimens may also decrease the time necessary for degradation, as a larger surface area-to-mass ratio may allow for more microbial access to the material at a time.

### Life cycle assessment results

Two Life Cycle Impact Assessment (LCIA) methodologies were employed using SimaPro software^51,52^ to calculate the environmental impacts of manufacturing both the plastic and compostable coffee pods. The first method, called CED, calculates the embodied energy within the supply chain. Embodied energy is the amount of energy that is embedded within the supply chain when all stages of the product (or process) from cradle to grave are accounted for^47–49^. This value is then summed up and represented with a unit called energy/amount (MJ/final part or MJ/kg in most cases). The second method follows the TRACI 2.1 v1.04 methodology, which employs a midpoint category LCIA method developed by the EPA to focus on US input parameters and locations^50^. The method focuses on 10 characterization factors to determine potential impacts from each material and process. These impact categories and their units include ozone depletion (kg CFC-11 eq), global warming (kg CO_2_ eq), smog (kg O_3_ eq), acidification (kg SO_2_ eq), eutrophication (kg N eq), carcinogenics (CTUh–comparative toxic units for humans), noncarcinogenics (CTUh), respiratory effects (kg PM2.5 eq–particulate matter), ecotoxicity (CTUe–comparative toxic units for environment), and fossil fuel depletion (MJ surplus). Details of TRACI calculations can be referenced from the work of Bare *et al*. within the updated user manual^58–60^. The reason for choosing the single-issue method alongside one that embodies multiple metrics is that energy, climate change, and cost assessment are cited as leading interests for organizations to potentially curb future emissions and in turn costs; however, it is pertinent not to look away from other hotspots that may arise as a result of other impact categories that are not evident—or in other words, burden shifting^61–63^.

**Figure 9:**
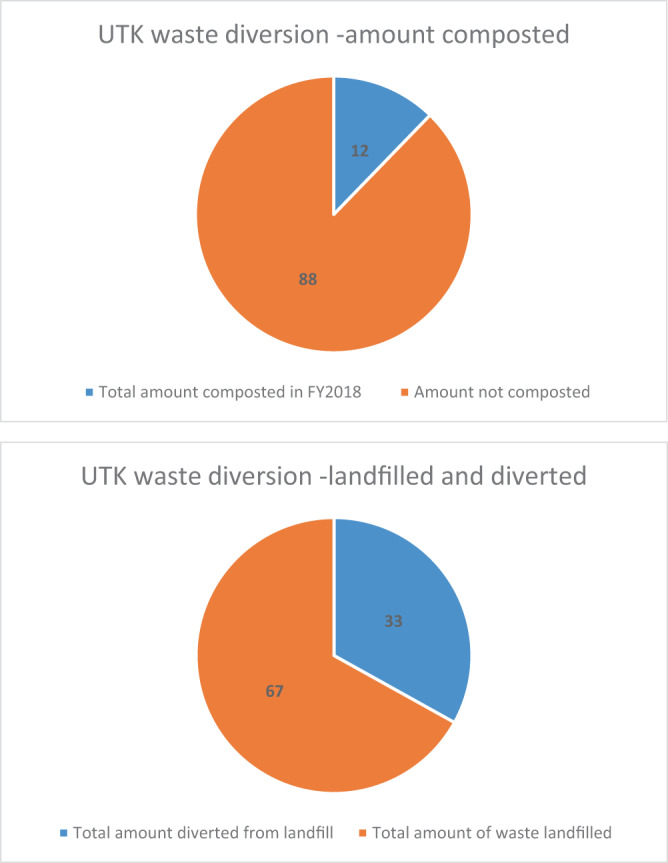
(**a**) UTK waste diversion representing amount composted in FY2018. (**b**) UTK waste diversion representing amount diverted from landfill in FY2018.

Two levels of calculation have been set up to estimate impacts per pod and impacts per year. The weight of one plastic and one compostable pod stands at 3.0213 g and 3.0622 g, respectively. Annual diversion rates, according to the sustainability office^64^ cite 12% as the amount of food waste (i.e., food, compost) and green waste (i.e., leaves, manure, wood chips, brush) composted on campus, with 67% being landfilled. The graphs in Fig. 9 represent waste diversion from the university for fiscal year (FY) 2018. The 12% from the chart on the left is part of the 33% in the chart on the right, which represents the amount of waste being diverted from landfills. Therefore, the difference of 21% is dedicated to recycling and other efforts on campus. The total amount of waste generated on campus in FY 2018 was 8711.34 metric tons (9602.61 short tons).

Considering the estimated 112,500 pods used per year on campus (based on the 750 pods per day estimation), the total weight of compostable pods equates to 344497.5 g (344 kg) annually, which may either be composted or landfilled. In terms of current plastic coffee pod consumption, this weight amounts to 339896.25 g (340 kg) with landfilling as the only disposal option. Referencing FY 2018, the environmental impacts of both plastic and compostable coffee pods have been highlighted in the following graphs while considering compost as an avoided product. This means that the impacts from creating compost as a result of the composting process have been used as credit to offset those impacts from the overall process. Compost is considered a value-added product. Therefore, 1 kg of compostable coffee pods input equates to the production of 1 kg of compost, with resultant compost impacts subtracted from the total impacts accrued.

#### Manufacturing each pod component

**Figure 10:**
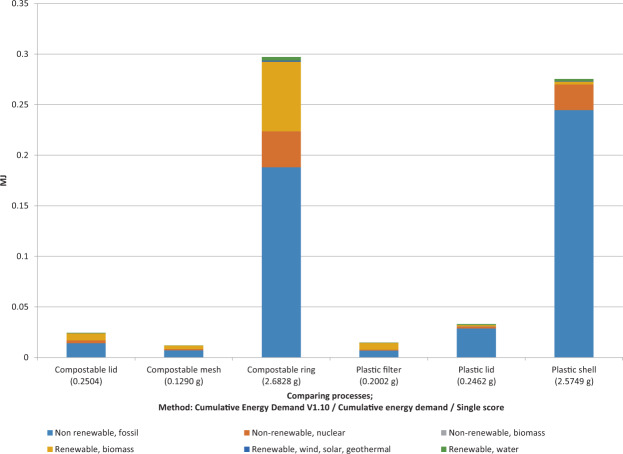
Embodied energy results of manufacturing compostable and plastic pod components.

**Figure 11:**
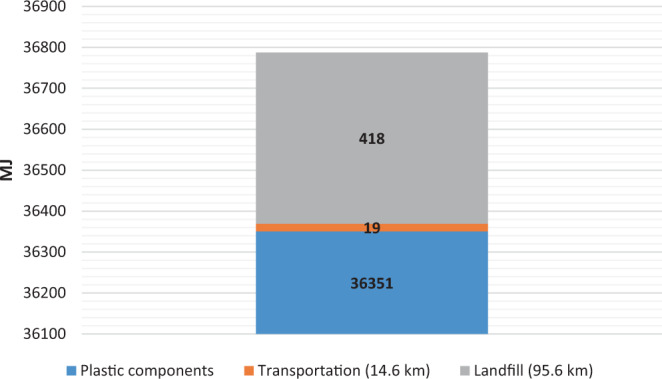
Embodied energy of annual plastic coffee pod consumption with current university scenario (FY 2018) from cradle to gate with transport and EOL options considering landfill scenario.

As preliminary analysis, the environmental impacts purely from production of various components of both kinds of coffee pods are shown in Figs. 10 and 11.

##### Energy Impact of Manufacturing Each Pod Component:

 The CED method was employed to understand the embodied energy of producing each component of the compostable and plastic pods. Figure 10 shows that producing the compostable ring of the pod consumes the most energy compared to any other components—0.297 MJ, whereas producing the compostable mesh consumes 0.012 MJ for both compostable and plastic coffee pods. The plastic shell has the highest consumption from amongst the plastic pod components, with 0.275 MJ, trailing by a mere 0.022 MJ for the compostable ring. The total embodied energy that goes into manufacturing a compostable coffee pod is 0.333 MJ/part (3.0622 g/pod) versus 0.323 MJ/part (3.0213 g/pod) for a plastic pod.

Although there is a minute difference of 0.01 MJ between the two pods being produced, the larger difference arises when the entire supply chain is taken into consideration, including procurement and EOL, which are represented from section Current University Scenario onward. Figure 10 shows that there is no significant energy difference between production of compostable versus plastic coffee pods. The energy differences in this case could also be attributed to the corresponding weights of components, with compostable pods being slightly heavier. Details of weights and other associated information can be accessed in Tables 2 and 3.

##### Environmental Impact of Manufacturing Each Pod Component:

 Figure 10 shows the manufacturing impacts from producing both types of pods in terms of characterization results from the TRACI method^50,59^ represented in Table 5. The highest values in each row are highlighted in bold and the least impact values are italicized for each category in subsequent tables. The compostable ring was found to dominate in 9 out of 10 categories except in the case of fossil fuel depletion, where the plastic shell dominates. This could be attributed to the source of a natural feedstock for compostable pods reducing fossil fuel impact. On closer analysis, higher impacts related to the compostable ring compared to those from plastic pod components may be associated with fertilizer input and leachate from harvesting the natural raw material from corn. This is considered within the process model, owing to the production of PLA, which is more dominant within the compostable ring process.

**Table 5:** TRACI characterization results of manufacturing compostable and plastic pod components.

Impact category	Unit	Compostable lid (0.2504 g)	Compostable mesh (0.1290 g)	Compostable ring (2.6828 g)	Plastic filter (0.2002 g)	Plastic lid (0.2462 g)	Plastic shell (2.5749 g)
Ozone depletion	kg CFC-11 eq	5.43E-11	*2.64E-11*	**2.41E-09**	3.18E-11	8.05E-11	1.91E-09
Global warming	kg CO2 eq	1.16E-03	5.80E-04	**1.48E-02**	*3.84E-04*	2.15E-03	9.70E-03
Smog	kg O3 eq	4.51E-05	*2.12E-05*	**5.57E-04**	2.54E-05	1.09E-04	4.24E-04
Acidification	kg SO2 eq	5.05E-06	2.45E-06	**6.26E-05**	*1.92E-06*	1.04E-05	3.63E-05
Eutrophication	kg N eq	5.54E-06	2.94E-06	**6.68E-05**	*1.19E-06*	4.26E-06	1.20E-05
Carcinogenics	CTUh	4.68E-11	2.25E-11	**5.71E-10**	*2.04E-11*	2.84E-10	3.27E-10
Noncarcinogenics	CTUh	1.15E-10	*4.92E-11*	**1.36E-09**	8.57E-11	3.57E-10	7.07E-10
Respiratory effects	kg PM2.5 eq	3.27E-07	*1.53E-07*	**3.90E-06**	4.85E-07	1.52E-06	2.60E-06
Ecotoxicity	CTUe	5.11E-03	2.56E-03	**6.03E-02**	*2.06E-03*	1.59E-02	2.38E-02
Fossil fuel depletion	MJ surplus	1.44E-03	*7.43E-04*	1.99E-02	8.02E-04	2.60E-03	3.21E-02

#### Current university scenario

Considering the current trend in coffee pod usage on campus, it is safe to assume that many pods used are made of plastic and end up in landfills. A local roasting and packaging company, 14.6 km away from the university, is considered as the site of procurement, and it is estimated that all pods eventually end up at the Meadow Branch Landfill in Athens, TN, 95.6 km away, along with the rest of campus waste that is not recycled or composted^65^. The municipal waste collection transportation data is included, taking into consideration a 21t municipal waste collection truck specific to the US, from the US-EI 2.2 dataset.

##### Energy impact of current university scenario:

The current university annual energy consumption from cradle to gate with transport and EOL options considering plastic pods add up to *36,788 MJ for 112,500 pods, each weighing 3.0213 g (340 kg total)*. The plastic filter, lid, and shell are stacked together as plastic components in Fig. 11. In this scenario, it is seen that the production of various plastic cup components dominates both the transportation and EOL stages: landfilling. The landfill process specifically considers the context of the United States, taking into account values for facility operation, closure, and leachate emissions. Landfill gas emissions being offset as a result of collection and conversion of gas to electricity is also taken into account, reducing some of its overall environmental impact.

##### Environmental impact of current university scenario:

**Figure 12:**
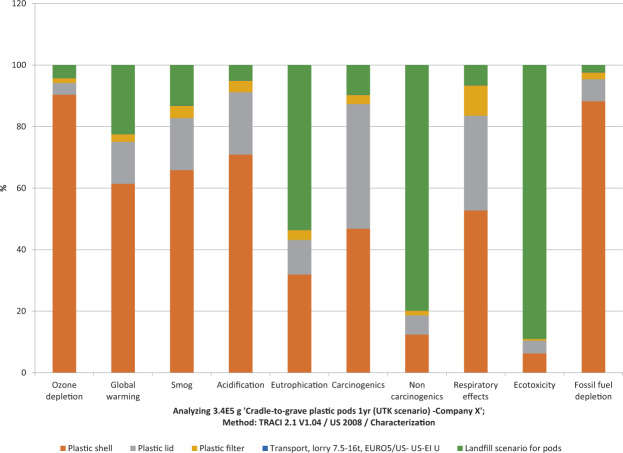
Characterization results of plastic and compostable coffee pods. Percentages represent the fraction of the emissions generated from each component and life cycle stage relative to the total emissions from the system. The percentages in each category add up to 100%.

 The annual environmental impact of disposing of 112,500 plastic coffee pods is analyzed in terms of manufacturing each plastic component, transportation (procurement only), and disposal (EOL transportation included). As such, it is seen from Fig. 12 that the process involved in producing the plastic shell (all components if stacked) has the highest impact in 7 out of 10 impact categories. Transportation was found to be least impactful on account of the coffee pods being procured from only 14.6 km away. This graph depicts the current landfill waste stream as problematic and in need of modification as eutrophication, noncarcinogenics, and ecotoxicity values peak with this process.

### Sensitivity analyses

Figure 12 and Table 6 indicate that the current method of disposal, landfilling, accounts for the most impact overall. Therefore, the Transportation sensitivity and End-of-life sensitivity sections explore how varying transportation distance and an alternative EOL disposal method (i.e., composting) affects the model overall.

**Table 6:** TRACI characterization values of annual plastic coffee pod consumption with current university scenario (FY2018) considering landfill as EOL scenario.

Impact category	Unit	Total	Plastic shell	Plastic lid	Plastic filter	Transport	Landfill scenario for pods
Ozone depletion	kg CFC-11 eq	2.37E-04	**2.15E-04**	9.05E-06	3.58E-06	*2.31E-07*	9.98E-06
Global warming	kg CO2 eq	1.78E + 03	**1.09E + 03**	2.42E + 02	4.33E + 01	*1.21E + 00*	3.99E + 02
Smog	kg O3 eq	7.25E + 01	**4.77E + 01**	1.23E + 01	2.86E + 00	*9.71E-02*	9.53E + 00
Acidification	kg SO2 eq	5.75E + 00	**4.08E + 00**	1.17E + 00	2.16E-01	*4.35E-03*	2.86E-01
Eutrophication	kg N eq	4.25E + 00	1.35E + 00	4.79E-01	1.34E-01	*1.20E-03*	**2.28E + 00**
Carcinogenics	CTUh	7.87E-05	**3.68E-05**	3.19E-05	2.30E-06	*5.82E-08*	7.57E-06
Noncarcinogenics	CTUh	6.42E-04	7.95E-05	4.02E-05	9.64E-06	*1.77E-07*	**5.12E-04**
Respiratory effects	kg PM2.5 eq	5.55E-01	**2.93E-01**	1.71E-01	5.46E-02	*3.62E-04*	3.63E-02
Ecotoxicity	CTUe	4.28E + 04	2.67E + 03	1.79E + 03	2.32E + 02	*3.68E + 00*	**3.81E + 04**
Fossil fuel depletion	MJ surplus	4.09E + 03	**3.61E + 03**	2.92E + 02	9.03E + 01	*2.40E + 00*	9.51E + 01

#### Transportation sensitivity

The impact related to procurement of the plastic pod was not high due to the presence of a local factory (14.6 km away) that roasted coffee and packaged plastic coffee pods in the area. This may not hold well while switching to compostable coffee pods. For this reason, a spreadsheet was created to map out all of the compostable coffee pod producers in the country and some crossing US–Canada border such as Ontario, Canada, to understand the best option for the university case while reducing impact from transporting these pods.

Primary keywords used for this search included “compostable coffee pod” and “compostable pods.” The options considered can be referenced from Supplementary Table S1. The values mentioned in this study are retail prices, implying that bulk pricing can be assumed to be even less. In addition, some product prices were found to fluctuate based on seasonal sales, promotions, and other factors. The published data was last updated and verified on March 11, 2019. According to the spreadsheet, the cheapest option mentioned was Company A with their pods priced at $0.35/pod shipped from San Francisco. The closest viable option was Company B sourced from Astoria, New York, 1165 km (724 mi) away at $0.82/pod. Because the first option was too far and the second too expensive, the next best option considering both distance and price was Company C from Toronto at a distance of 1179.7 km (733 mi) with a price of $0.49/pod, which has been considered optimum for modeling.

For distance information, the source taken for reference here comes from AASHE STARS (The Association for the Advancement of Sustainability in Higher Education Sustainability Tracking, Assessment & Rating System), a voluntary, self-reporting framework for single-ingredient products stating: *‘All production, processing, and distribution facilities must be within a 250 mile (400 kilometre) radius of the institution. This radius is extended to 500 miles (800 kilometres) for meat (i.e., beef, lamb, pork, game)*^66^.’ Therefore, the chosen distances for sensitivity analysis are 0 km (0 mi), 402.3 km (250 mi), 804.7 km (500 mi), and 1609.3 km (1000 mi) as the upper limit, representing neither local nor sustainable sourcing^67,68^. In addition, the proposed distance of 1179.7 km (733 mi) was selected as the average distance among the compostable coffee pod distributors.

##### Energy impact of transportation sensitivity:

This step involved inspecting various distances to understand the impact of transport on the overall model. All scenarios in Table 7 incorporate landfill distance as 95.6 km and compost site distance as 6.3 km. In this case, only the procurement distance was varied to see changes in overall embodied energy.

**Table 7:** Transportation sensitivity analysis with CED results for varying procurement distances based on annual consumption.

Label	Baseline (MJ)	Total (MJ)	Rank	Observed change
Compost scenario compostable pods only (UTK avoided product -1 yr) -Company C Scenario	3.68E + 04	3.90E + 04	4	+6.1%
**Compost scenario compostable pods only (UTK avoided product -1 yr)** **0 km (0 mi)**		**3.75E + 04**	**12**	**+1.9%**
Compost scenario compostable pods only (UTK avoided product -1 yr) 402.3 km (250 mi)		3.80E + 04	9	+3.3%
Compost scenario compostable pods only (UTK avoided product -1 yr) 804.7 km (500 mi)		3.85E + 04	7	+4.8%
Compost scenario compostable pods only (UTK avoided product -1 yr) 1609.3 km (1000 mi)		3.96E + 04	2	+7.6%
Landfill scenario compostable pods only (UTK -1yr) -Company C: 1179.7 km (733 mi)		3.95E + 04	3	+7.3%
**Landfill scenario compostable pods only (UTK -1yr)** **0 km (0 mi)**		**3.79E + 04**	**10**	**+3.1%**
Landfill scenario compostable pods only (UTK -1yr) 402.3 km (250 mi)		3.84E + 04	8	+4.5%
Landfill scenario compostable pods only (UTK -1yr) 804.7 km (500 mi)		3.90E + 04	5	+5.9%
Landfill scenario compostable pods only (UTK -1yr) 1609.3 km (1000 mi)		4.00E + 04	1	+8.8%
Plastic pods 1 yr (UTK scenario) -Company X: 14.6 km (9.1 mi)		3.68E + 04	14	0%
**Plastic pods 1 yr (UTK scenario)** **0 km (0 mi)**		**3.68E + 04**	**15**	**-0.1%**
Plastic pods 1 yr (UTK scenario) 402.3 km (250 mi)		3.73E + 04	13	+1.4%
Plastic pods 1 yr (UTK scenario) 804.7 km (500 mi)		3.78E + 04	11	+2.8%
Plastic pods 1 yr (UTK scenario) 1609.3 km (1000 mi)		3.89E + 04	6	+5.6%

All control distances (0 km) are highlighted in bold. Company C is the suggested scenario, with Company X being the currently used conventional pod scenario.

Fifteen scenarios were compared for this analysis, with varying distances of 0 km (0 mi), 402.3 km (250 mi), 804.7 km (500 mi), 1609.3 km (1000 mi), current plastic procurement scenario (14.6 km), and the proposed compostable pod scenario (1179.7 km). Compared to the baseline scenario of plastic pods being transported for only 14.6 km before being landfilled, the only other distance that would consume less energy is the control for the plastic pods, where procurement distance is 0 km. Control distances are in bold as these are not realistic options, rather more so for reference.

When these values are ranked, it is seen that energy consumption is least with the current baseline, where plastic pods are landfilled and procured up to an 804.7 km (500 mi) radius compared to compostable pods being landfilled or composted locally. It is interesting to note that the proposed optimum compostable pod here would cause a 6.1% increase in the overall embodied energy of the supply chain compared to the current plastic pod use-and-throw system. This may be attributed to the energy embedded within harvesting natural systems versus those encompassing a purely extraction-based system, or the fact that operations related to composting are much more intensive than those at a landfill. As this is a single-issue method that focuses on embodied energy as the only assessed environmental impact, this does not give a holistic picture of other impacts involved in the system, which is why exploring other LCA methods such as TRACI would aid in checking for such variation.

##### Environmental impact of transportation sensitivity:

 In this case, the same assumptions as in section Energy impact of transportation sensitivity are taken while checking for the resultant environmental impacts.

Tables 8, 9 shows that the overall impacts of a product cannot be obtained from any single-issue method, such as CED. Although the embodied energy of landfilling plastic pods up to an 804.7 km (500 mi) radius procurement distance was found to be less compared to composting or landfilling compostable pods (Table 7), the other impacts reinforce a different conclusion. A review of the TRACI method’s 10 environmental impact categories shows that compostable pods that are landfilled have the highest impact overall (8 out of 10) except for respiratory effects and fossil fuel depletion, for which the plastic pods being landfilled have the highest impact. A process contribution analysis showed that the compostable ring was the main cause for the spike in most of these flows. The decrease in fossil fuel depletion–related impacts can be attributed to the modeling of the PLA process, which contains less fossil fuel input compared to PP. Higher respiratory effects and fossil depletion are seen from landfilling plastic pods when the procurement distance extends to 1609.3 km (1000 mi), which may be due to transportation distance playing a role in the overall impact, with more fossil fuel consumption and particulate pollution resulting from this scenario. The least impacts come from the compostable pods being composted in 7 out of 10 categories, with the exception of ozone depletion, acidification, and eutrophication. It is projected that the proposed switch from the current Company X plastic pods to Company C compostable pods, procured from 1179.7 km away and composted on campus, could result in a decrease in 5 out of 10 impact categories compared to the current scenario. There will be a slight increase in carcinogenics (2.44%) and respiratory effects (6.18%) and significant increases in noncarcinogenics (71%), ecotoxicity (81.31%), and fossil fuel depletion (34.49%). Because the CED method calculated a 6% increase in terms of embodied energy earlier, the fossil fuel depletion category, which looks into MJ surplus, can be ignored.

**Table 8:** Transportation sensitivity analysis with TRACI characterization results for varying procurement distances based on annual consumption. Baseline category refers to the suggested Company C scenario in row 1.

Impact category	Ozone depletion	Global warming	Smog	Acidification	Eutrophication	Carcinogenics	Noncarcinogenics	Respiratory effects	Ecotoxicity	Fossil fuel depletion
Unit	kg CFC-11 eq	kg CO2 eq	kg O3 eq	kg SO2 eq	kg N eq	CTUh	CTUh	kg PM2.5 eq	CTUe	MJ surplus
Compost scenario compostable pods only (UTK avoided product -1 yr) –Company C Scenario 1179.7 km (733 mi)	2.99E-04	1.87E + 03	7.44E + 01	7.95E + 00	8.55E + 00	7.67E-05	1.86E-04	5.21E-01	7.99E + 03	2.68E + 03
Compost scenario compostable pods only (UTK avoided product -1 yr) 0 km (0 mi)	2.80E-04	*1.77E + 03*	*6.64E + 01*	7.60E + 00	8.45E + 00	*7.20E-05*	*1.71E-04*	*4.91E-01*	*7.69E + 03*	*2.48E + 03*
Compost scenario compostable pods only (UTK avoided product -1 yr) 1609.3 km (1000 mi)	3.06E-04	1.90E + 03	7.72E + 01	8.08E + 00	8.58E + 00	7.85E-05	1.91E-04	5.32E-01	8.10E + 03	2.75E + 03
Compost scenario compostable pods only (UTK avoided product -1 yr) 402.3 km (250 mi)	2.87E-04	1.80E + 03	6.91E + 01	7.72E + 00	8.48E + 00	7.36E-05	1.76E-04	5.01E-01	7.79E + 03	2.55E + 03
Compost scenario compostable pods only (UTK avoided product -1 yr) 804.7 km (500 mi)	2.93E-04	1.84E + 03	7.18E + 01	7.84E + 00	8.51E + 00	7.52E-05	1.81E-04	5.11E-01	7.90E + 03	2.62E + 03
Landfill scenario compostable pods only (UTK -1yr) -Company C Scenario	3.09E-04	2.37E + 03	8.77E + 01	8.53E + 00	1.09E + 01	8.44E-05	7.05E-04	5.59E-01	4.65E + 04	2.77E + 03
Landfill scenario compostable pods only (UTK -1yr) 0 km (0 mi)	2.90E-04	2.27E + 03	7.98E + 01	8.17E + 00	1.08E + 01	7.97E-05	6.90E-04	5.29E-01	4.62E + 04	2.58E + 03
Landfill scenario compostable pods only (UTK -1yr) 1609.3 km (1000 mi)	**3.16E-04**	**2.40E + 03**	**9.06E + 01**	**8.66E + 00**	**1.09E + 01**	**8.61E-05**	**7.10E-04**	5.69E-01	**4.66E + 04**	2.85E + 03
Landfill scenario compostable pods only (UTK -1yr) 402.3 km (250 mi)	2.97E-04	2.30E + 03	8.25E + 01	8.29E + 00	1.08E + 01	8.13E-05	6.95E-04	5.39E-01	4.63E + 04	2.65E + 03
Landfill scenario compostable pods only (UTK -1yr) 804.7 km (500 mi)	3.03E-04	2.34E + 03	8.52E + 01	8.42E + 00	1.08E + 01	8.29E-05	7.00E-04	5.49E-01	4.64E + 04	2.71E + 03
Plastic pods 1 yr (UTK scenario) -Company X 14.6 km (9.1 mi)	2.37E-04	1.78E + 03	7.25E + 01	5.75E + 00	4.25E + 00	7.87E-05	6.42E-04	5.55E-01	4.28E + 04	4.09E + 03
Plastic pods 1 yr (UTK scenario) 0 km (0 mi)	*2.37E-04*	1.77E + 03	7.24E + 01	*5.75E + 00*	*4.24E + 00*	7.86E-05	6.42E-04	5.55E-01	4.28E + 04	4.08E + 03
Plastic pods 1 yr (UTK scenario) 402.3 km (250 mi)	2.44E-04	1.81E + 03	7.51E + 01	5.87E + 00	4.28E + 00	8.02E-05	6.47E-04	5.65E-01	4.29E + 04	4.15E + 03
Plastic pods 1 yr (UTK scenario) 804.7 km (500 mi)	2.50E-04	1.84E + 03	7.78E + 01	5.99E + 00	4.31E + 00	8.18E-05	6.51E-04	5.75E-01	4.30E + 04	4.22E + 03
Plastic pods 1 yr (UTK scenario) 1609.3 km (1000 mi)	2.63E-04	1.91E + 03	8.31E + 01	6.23E + 00	4.38E + 00	8.50E-05	6.61E-04	**5.95E-01**	4.32E + 04	**4.35E + 03**
**Percent change to highest impact from suggested option**	**6%**	**29%**	**22%**	**9%**	**28%**	**12%**	**283%**	**14%**	**484%**	**62%**
**Percent change to least impact from suggested option**	**-21%**	**-5%**	**-11%**	**-28%**	**-50%**	**-6%**	**-8%**	**-6%**	**-4%**	**-7%**
**Percent change from baseline to suggested**	**-26%**	**-5%**	**-3%**	**-38%**	**-101%**	**2%**	**71%**	**6%**	**81%**	**34%**

**Table 9:** Observed changes with varying EOL compost-to-landfill ratios in terms of CED results compared to baseline scenario based on annual consumption.

Scenario description	Source	Baseline (MJ)	Result (MJ)	Observed change
Plastic pod	Plastic pod (14.6 km) 100% LANDFILL	3.68E + 04	3.68E + 04	0
	Plastic pod (1179.7 km) 100% LANDFILL		3.83E + 04	+4.10%
Compostable pod (1179.7 km)	*100% COMPOST* *0% LANDFILL*		*3.90E + 04*	*+6.10%*
	*80% COMPOST* *20% LANDFILL*		*3.91E + 04*	*+6.34%*
	50% COMPOST 50% LANDFILL		3.92E + 04	+6.69%
	20% COMPOST 80% LANDFILL		3.94E + 04	+7.04%
	**0% COMPOST** **100% LANDFILL**		**3.95E + 04**	**+7.27%**

#### End-of-life sensitivity

Sensitivity to EOL scenarios allows for consideration of differing landfill-to-compost ratios for disposal. Ratios assessed are 20:80, 50:50, and 80:20 for the proposed compostable cups. This is compared with the current situation of plastic pods landfilled 100% for comparison.

##### Energy impact of EOL sensitivity:

**Figure 13:**
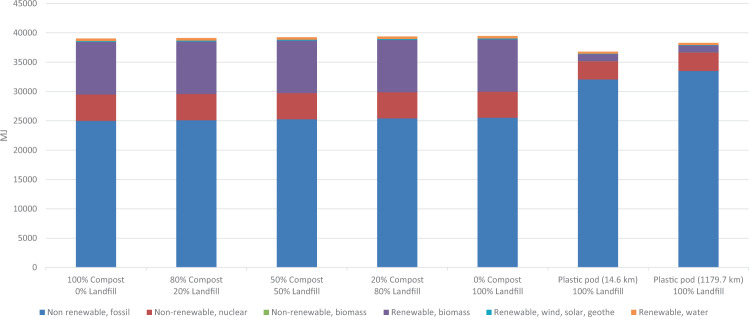
EOL sensitivity analysis with CED results for varying compost-to-landfill ratios based on annual consumption.

 Figure 13 and Table 4 show how the overall model varies when waste diversion ratios are altered. For the sake of comparison, the plastic pod was also checked with transportation distance equal to the newly proposed compostable pod (i.e., 1179.7 km) for comparison with the same procurement distance. This shows a 4.1% increase in terms of embodied energy with respect to the current plastic pod scenario displaying the impact of transportation in the overall model. However, this number is still less than the embodied energy of all other composting scenarios.

Similar to the previous sensitivity with transport, the landfilling and composting of compostable pods shows a higher embodied energy than landfilling plastic pods from the current scenario. Even with the hypothetical best-case scenario that all compostable pods end up composted, it still equates to a 6.1% overall increase in embodied energy. One significant difference between the two types of pods is the difference in terms of fossil fuel consumption and biomass utilization from the sources of raw materials.

##### Environmental impact of EOL sensitivity:

 Table 10 represents the same model as in section Energy impact of EOL sensitivity but with varying waste diversion rates in terms of environmental impact categories taken from the TRACI method. Here, it is seen that landfilling 100% of either plastic or compostable pod leads to greater impacts overall except in the case of the current scenario in which the transportation distance of ~15 km plays a role.

**Table 10:** Observed changes with varying EOL compost-to-landfill ratios in terms of TRACI characterization results based on annual consumption.

Impact category	Ozone depletion	Global warming	Smog	Acidification	Eutrophication	Carcinogenics	Noncarcinogenics	Respiratory effects	Ecotoxicity	Fossil fuel depletion
Unit	kg CFC-11 eq	kg CO2 eq	kg O3 eq	kg SO2 eq	kg N eq	CTUh	CTUh	kg PM2.5 eq	CTUe	MJ surplus
Compost scenario compostable pods only (UTK avoided product -1 yr) COMP100-LAND0	2.99E-04	1.87E + 03	7.44E + 01	7.95E + 00	8.55E + 00	*7.67E-05*	*1.86E-04*	*5.21E-01*	*7.99E + 03*	*2.68E + 03*
Compost scenario compostable pods only (UTK avoided product -1 yr) COMP20-LAND80	3.07E-04	2.27E + 03	8.50E + 01	8.41E + 00	1.04E + 01	8.29E-05	6.01E-04	5.51E-01	3.88E + 04	2.76E + 03
Compost scenario compostable pods only (UTK avoided product -1 yr) COMP50-LAND50	3.04E-04	2.12E + 03	8.10E + 01	8.24E + 00	9.71E + 00	8.06E-05	4.45E-04	5.40E-01	2.73E + 04	2.73E + 03
Compost scenario compostable pods only (UTK avoided product -1 yr) COMP80-LAND20	3.01E-04	1.97E + 03	7.70E + 01	8.07E + 00	9.01E + 00	7.83E-05	2.89E-04	5.28E-01	1.57E + 04	2.70E + 03
Landfill scenario compostable pods only (UTK -1yr) LAND100-COMP0	**3.09E-04**	**2.37E + 03**	**8.77E + 01**	**8.53E + 00**	**1.09E + 01**	**8.44E-05**	**7.05E-04**	5.59E-01	**4.65E + 04**	2.77E + 03
Plastic pods 1 yr (UTK scenario) -Company X	*2.37E-04*	*1.78E + 03*	*7.25E + 01*	*5.75E + 00*	*4.25E + 00*	7.87E-05	6.42E-04	5.55E-01	4.28E + 04	4.09E + 03
Plastic pods 1 yr (UTK scenario) -TEST 1179.7 km (733 mi)	2.56E-04	1.87E + 03	8.03E + 01	6.10E + 00	4.34E + 00	8.33E-05	6.56E-04	**5.84E-01**	4.31E + 04	**4.28E + 03**
**Percent change from least to highest impact**	**30%**	**33%**	**21%**	**48%**	**156%**	**10%**	**280%**	**12%**	**482%**	**60%**

For this purpose, a TEST model was run with procurement distance matching that of the suggested compostable pods (1179.7 km) to see how impacts would vary. The results come close to those mentioned for the 100% composting option and yet maintain an advantage with respect to the acidification and eutrophication levels. This could be owing to the natural components requiring fertilizer input, not being inert, and releasing leachates as a result of growing corn for PLA production, which is embedded within the supply chain.

Although composting 100% of the pods seems to be the best option, realistically, this may not be feasible due to several other reasons such as failure to collect all pods or users failing to segregate waste properly. Composting compostable pods is seen as the preferable option while achieving higher percentages of composting ability.

### Projected scenarios

Coffee itself has not been taken into consideration in any of the models due to complexity in terms of the coffee source, harvesting method, species, roasting processing, and other associated parameters including how caffeine behaves within the compost site. Within SimaPro, coffee green bean production considers averages of Arabica and Robusta beans but does not distinguish organic standards. Li^16^ has done a simulation with untreated wood waste in which 20% moisture taken as proxy for coffee ground waste to see how it would impact the overall supply chain. If wet weights were to be included, the results would be very different. In scenarios presented within the next two sections, the wet weight constitutes tap water that would remain in the pods while extracting coffee during usage. The wet weight values were obtained from weighing pods after usage. Because this stays within the coffee pod until disposal, this model only considers the final wet weight impact of transporting all of these pods to either the landfill or compost site.

#### Energy and environmental impact of coffee pods with wet weight and distances & wet weight and no distance

This considers models with wet weights of both types of pods with their current procurement distances compared to results exported with wet weights of the same pods considering no procurement distance to differentiate between the current and proposed scenarios and to establish a control with no distance, similar to previous sensitivity models. This would be useful within the context of assessing disposal costs for the used pods from the facilities point of view. The total annual water weight adds up to 933.75 kg and 1046.250 kg for plastic and compostable pods, respectively. The difference in weight between both types of used pods adds equals 483.75 kg, which is substantial while assessing energy, environmental impacts, and disposal costs. The trends here are similar to those reflected in the previous analyses in which transportation and EOL waste streams are involved. Table 11 highlights the fact that, in terms of embodied energy and other environmental impacts, the landfilled compostable pods exhibit the highest impacts. In terms of only embodied energy, landfilled plastic pods present the least impacts compared to landfilling compostable pods, which have the worst impacts unless composted. However, it should be noted that this case does not take into account the value of savings from composting versus landfilling—$14,553–$30,303—or avoiding the cost of purchasing fresh compost for campus farms, gardens, and landscaping.

**Table 11:** CED and TRACI results for projected wet weight scenarios with current and control distances based on annual consumption.

Impact category	Unit	Compost scenario compostable pods only (UTK avoided product -1 yr) -Company C Scenario + wet weight	Compost scenario compostable pods only (UTK avoided product -1 yr) + wet weight + no distance	Landfill scenario compostable pods only (UTK -1yr) -Company C Scenario + wet weight	Landfill scenario compostable pods only (UTK -1yr) + wet weight + no distance	Plastic pods 1 yr (UTK scenario) -Company X + wet weight	Plastic pods 1 yr (UTK scenario) + wet weight + no distance	Percent difference between best and worst values
Ozone depletion	kg CFC-11 eq	1.40E-03	1.31E-03	**1.48E-03**	1.39E-03	8.92E-04	*8.91E-04*	66%
Global warming	kg CO2 eq	8.43E + 03	7.97E + 03	**1.23E + 04**	1.18E + 04	7.56E + 03	*7.56E + 03*	63%
Smog	kg O3 eq	3.36E + 02	2.99E + 02	**4.39E + 02**	4.02E + 02	2.90E + 02	*2.90E + 02*	51%
Acidification	kg SO2 eq	3.63E + 01	3.46E + 01	**4.07E + 01**	3.91E + 01	2.17E + 01	*2.17E + 01*	87%
Eutrophication	kg N eq	3.99E + 01	3.94E + 01	**5.78E + 01**	5.73E + 01	2.17E + 01	*2.17E + 01*	166%
Carcinogenics	CTUh	3.58E-04	3.36E-04	**4.17E-04**	3.95E-04	3.07E-04	*3.07E-04*	36%
Non carcinogenics	CTUh	8.67E-04	*8.00E-04*	**4.87E-03**	4.80E-03	3.74E-03	3.74E-03	509%
Respiratory effects	kg PM2.5 eq	2.43E + 00	2.29E + 00	**2.72E + 00**	2.58E + 00	2.12E + 00	*2.12E + 00*	28%
Ecotoxicity	CTUe	3.74E + 04	*3.60E + 04*	**3.34E + 05**	3.33E + 05	2.60E + 05	2.60E + 05	828%
Fossil fuel depletion	MJ surplus	1.25E + 04	*1.16E + 04*	1.33E + 04	1.23E + 04	**1.51E + 04**	1.51E + 04	31%
CED Result	MJ	1.82E + 05	1.75E + 05	**1.86E + 05**	1.78E + 05	1.35E + 05	*1.35E + 05*	37%

#### Energy and environmental impact of coffee pods with same weights and no distance

Due to the fact that, in many of the cases mentioned previously, the weight of compostable pods is higher than the plastic ones, inherently slightly more impacts will be felt within certain categories. Therefore, to account for a direct comparison between both plastic and compostable pods with equal weightage, the model represented in Table 12 compares the EOL processes without dealing with any difference in terms of weight or transport distance. This would be the closest 1:1 comparison for both products that can be applied for reference.

**Table 12:** CED and TRACI results for projected wet weight scenarios with equal pod weights based on annual consumption.

Impact category	Unit	Compost scenario compostable pods only (UTK avoided product -1 yr) 0 km	Landfill scenario compostable pods only (UTK -1yr) 0 km	Plastic pods 1 yr (UTK scenario) 0 km	Percent change between best and worst
Ozone depletion	kg CFC-11 eq	8.14E-04	**8.43E-04**	*6.98E-04*	21%
Global warming	kg CO2 eq	*5.13E + 03*	**6.58E + 03**	5.22E + 03	28%
Smog	kg O3 eq	*1.93E + 02*	**2.32E + 02**	2.13E + 02	20%
Acidification	kg SO2 eq	2.21E + 01	**2.37E + 01**	*1.69E + 01*	40%
Eutrophication	kg N eq	2.45E + 01	**3.13E + 01**	*1.25E + 01*	150%
Carcinogenics	CTUh	*2.09E-04*	2.31E-04	2.31E-04	11%
Non carcinogenics	CTUh	*4.97E-04*	**2.00E-03**	1.89E-03	303%
Respiratory effects	kg PM2.5 eq	*1.43E + 00*	1.54E + 00	1.63E + 00	14%
Ecotoxicity	CTUe	*2.23E + 04*	**1.34E + 05**	1.26E + 05	501%
Fossil fuel depletion	MJ surplus	*7.20E + 03*	7.49E + 03	1.20E + 04	67%
CED Result	MJ	1.09E + 05	**1.10E + 05**	*1.08E + 05*	2%

^*^The weight taken for each scenario is 1 metric ton (1000 kg).

In terms of embodied energy and characterized TRACI values, once again the landfilled compostable pods exhibit the highest impacts. The key takeaway here is that the landfilled plastic pods contain only 3 out of 10 of the minimal impacts with respect to ozone depletion, acidification, and eutrophication, complying with the reasons mentioned previously. They also present three of the highest impacts in terms of carcinogenics, respiratory effects, and fossil fuel depletion. Therefore, analyzing the impacts of both kinds of pods when their weights are equal, the compostable pods that are composted have the least amount of impacts in 7 out of 10 categories.

## Interpretation

It is to be noted that certain value choices and judgments present in this study may have affected the results of the LCA:

1. Numbers determined as least estimate values while assessing blueprints based on office, lab, kitchen, dorm, and classroom space, which can vary due to system updates and approach over time. The current method was established in November 2018 and considers one pod per coffee machine.

2. Model for composting and landfilling process with set distances.

3. Proxies for raw materials that were not available readily in the software.

4. Exclusion/inclusion of credits in the EOL stage with avoided product could change the results, especially for compost by a slight margin.

## Discussion

Moving forward, it may be possible to set up a contract with an official coffee supplier for the university’s single-serve coffee machines and all official university functions. This will undoubtedly require greater stock of coffee and result in a decrease of cost due to bulk purchasing. Because the public tends to desire convenience over sustainable practices, compostable pods may be an appropriate middle ground between the general university community and those wishing to implement more sustainable habits on campus. With easy disposal methods conducive to channeling into an established local waste stream on campus, along with the relatively low costs associated with both purchasing and sustainable disposal, using compostable coffee pods can prove to be effective in reducing environmental and financial burdens. Other initiatives that can be suggested alongside this new institutional change is the establishment of “tiny trash” cans for storage of used compostable coffee pods prior to a bigger compost collection. The tiny trash concept (small trash bins attached to recycling receptacles in offices for discarding small non-recyclable materials) has proven to decrease landfill waste on campus and could be extended for coffee pods^59,69^. In addition, there is always scope for improvement in terms of innovation, new methods for brewing coffee, single-serve coffee pod design, and development of mechanisms for storage and disposal of the grounds. As water has to be refilled, it may be possible to reengineer the machines for waste saving and convenience.

Li^16^ mentions compostable coffee pod producers stating that these pods have the capability of breaking down within twelve weeks in a municipal composting facility after being subjected to strict testing according to standards. The results from this thesis looks at comparing the environmental impacts of a plastic, aluminum, and biodegradable coffee pods (their reference to the compostable ones) within the context of Ontario, Canada –which state that the biodegradable ones embodied less environmental impacts with respect to GWP, acidification, and respiratory effects but rather the sustainability factor also depends on the diversion from landfills, which is one of the reasons for stressing the importance of procurement distance and EOL methods in this case study. They also reference a traditional drip-coffee method where it was found that retail packaging was much higher for single serve pods due to the use of more material.

This particular study was done with the intention of a typical US university setting in mind. When comparing such a scenario to larger entities such as a municipality, town, or country, or smaller units such as a residential building, an extra layer of complexity is added due to the need to locate composting facilities within a reasonable distance. It is important to take note of local legislative policies and frameworks that support the effective operation and management of such operations and those that incentivize the process^70^.

A report compiled by BioCycle in 2017 stated that the United States had 4,713 active industrial composting facilities^71^. Bioselect has also mapped out industrial food composting facilities^72^. Diversion mandates and disposal bans were found to be the main reasons driving this industry forward with mostly state funding and public–private partnerships propelling such facilities. Websites such as findacomposter.com allow users to search for the closest industrial composting facility^73^. Therefore, with a combination of available tools, combined with expert opinion and collaboration with organizations integral to this field, questions such as these may be addressed at a range of economies of scale.

Availability of metrics in addition to effective communication from municipalities regarding outlets for industrial composting are important steps for success. The impacts depend on the availability of infrastructure for regular collection, sorting mechanisms, and processing within the area of waste generation. Because LCAs cannot offer a one-size-fits-all solution, potential points of variation from this study that may be applicable to others are:

1. Cost of waste disposal methods due to the proximity of local landfills or community compost sites.
2. Motivation behind composting (i.e., state mandates, incentives, social enterprises).
3. Procurement distance of products.
4. Baselining impacts of incumbent products/processes for comparison with alternative options; Li^16^ considered Canadian statistics, where it was estimated that the city would consume 2 billion coffee pods per year with an average weight of 15 g per pod.
5. Cost–benefit analysis to determine the economic feasibility of such operations.
6. Collection, sorting, screening, and separation of contaminants.
7. Community participation–whether it be active involvement from students, constituents, or industries, active support is integral for composting work to come to fruition and thrive.

In this sense, it is assumed that the base materials for plastic versus compostable pods remain somewhat similar to those examined within this study. One assumption is that most environmental impacts would be similar to the ones noted in this case for a representative baseline. However, the transportation distance in terms of procurement and EOL will require careful investigation in order to determine the best possible outcome.

In one study^74^ that compared three types of coffee preparation methods (drip, French press, and pod-style), landfilling as biodegradable waste was considered as the disposal method for coffee grounds and filter as biodegradable and plastic and aluminum was considered as inert. Recycling and composting was mentioned as a possibility but not explored in this study. However, one of the key takeaways was that, due to varying amounts of coffee grounds being used for preparing coffee with different methods, this may cause burden shifting in terms of impacts. With the TRACI method, single-serve pods were found to have a lower environmental impact compared to the conventional drip filter method. Schlesinger concludes that the most sustainable way of consuming coffee would be to purchase shade-grown coffee, brewing it by French press, and disposing of the coffee grounds in compost, as caffeine that is not removed by sewage treatment (varying from 2–30%) may contaminate water systems, which in turn has an effect on ecosystems. It was determined that the type of coffee equipment used as well as the mechanism used to keep the coffee warm are integral to energy consumption. While traditional brewers consume more energy as the entire pot must be kept heated, single-pod brewing machines are better when a single pod is used and the machine switched off, but they fare the worst in terms of energy consumption when left on standby^75^. Yang *et al*. mention previous efforts aimed at combusting used plastic coffee pods for energy supply with residual ash used for cement production due to it being a mixed-material product with inability to separate components. The authors also mentioned the plastic pods being incinerated for use as an alternative to fossil fuel energy within the process. In this scenario, the project was found to be successful due to the coffee service delivering and recovering the used pods specifically with this intention. In their study, crude bio-oil was obtained through a liquefaction process employing a water-ethanol mixture (in 50/50 v/v); it was found that the crude oil was mostly derived from the biomass, with plastic components having little contribution. Alkaline catalysts such as NaOH were found to have significantly decomposed the feedstock, enhancing bio-oil production and liquefaction efficiency, and acidic ones were found to have a negative impact of decreasing production. However, successful application of this technology was not presented^76^. Some pilot decentralized composting facilities have had success, which have given scope for successive establishments; one in Dhaka, Bangladesh, stands as a testament to the feasibility of such operations, leading to the establishment of 14 new composting facilities within the city^77^. These studies were found to have some overlap in terms of product systems, processes, and disposal mechanisms. Nevertheless, they were still very different in terms of system boundaries, methodologies, and scale, which only adds fuel to the debate regarding not having an apples-to-apples comparison for LCA studies.

## Conclusion

This paper takes on a university case of estimating both energy and environmental impacts of consuming plastic and compostable coffee pods on campus. The values mentioned are low estimates. Although in all of these cases, landfilling plastic pods fares best in terms of embodied energy, while looking at 10 other environmental impact categories, that is not the case. This proves that, to gain a holistic understanding of impacts related to a certain product, a single-issue method does not suffice. From the combination of lower impacts related to composting and lower cost in terms of net purchase and disposal showing cost savings up to 21% while switching to composting optimized distance compostable pods—rather than landfilling conventional plastic pods—it is concluded that the compostable pods have the least burden on the environment and the university budget alike. This number may show more savings if the cost of avoiding the purchase of fresh compost is taken into consideration. The study can be taken as a reference document for other organizations to understand the impacts of several other products that are consumed on a daily basis to establish respective baselines, highlight environmental hotspots, and offer environmentally conscious solutions.

The university can effectively drive institutional change by incorporating these results into the university’s current climate neutrality goal for 2061, if not sooner^78^. The present study provides a framework for other academic institutions to consider coffee pods disposal within their own setting. This study considers both plastic and compostable coffee pod options with detailed analysis of viable disposal streams while exploring feasible options in the community. These results differ from others in the sense that a traditional LCA alongside experimental data has been recorded to show the success within a composting facility from the commencement to conclusion. These values were utilized to fit the scenario encountered at the university to highlight environmental hotspots and to determine the best course of action moving forward in terms of procuring coffee from the closest vendors that produce compostable coffee pods as well as the best pricing options to cater to the triple bottom line (people, planet, and profit).

### LCA Critical Review

This LCA study is compliant with ISO 14040 and ISO 14044. The critical review was conducted by LCA certified professionals Kovvali Manasa Rao and Brad McAllister of WAP Sustainability Consulting, Chattanooga, Tennessee.

### Disclaimer

The information, data, or work presented herein was funded in part by an agency of the United States Government. Neither the United States Government nor any agency thereof, nor any of their employees, makes any warranty, express or implied, or assumes any legal liability or responsibility for the accuracy, completeness, or usefulness of any information, apparatus, product, or process disclosed, or represents that its use would not infringe privately owned rights. Reference herein to any specific commercial product, process, or service by trade name, trademark, manufacturer, or otherwise does not necessarily constitute or imply its endorsement, recommendation, or favoring by the United States Government or any agency thereof. The views and opinions of authors expressed herein do not necessarily state or reflect those of the United States Government or any agency thereof.

### Appendix

University coffee pod usage estimation

The portal contains infrastructure to split the categorical areas of interest apart and view the respective floors, as well as locate other identifying information such as current occupancy, size, location, and department. Viewing the data in a tabulated form allows the user to count the floors at which known single-serve coffee machines may occur due to a satisfactory office space, self-serve dining area, meeting room, or residence hall. The user may differentiate office spaces to include positions permanently occupied or known to have continued residence throughout a semester; this may be checked by viewing the occupancy and department data located on the same page. Satisfying conditions within section Pod usage within the university campus based on the various areas mentioned, the spaces with respective counts are as follows: faculty (75), library faculty (16), staff (85), clerical (213), and visitor (106). For any office assumed to have a single-serve coffee machine on a floor, no more values were added for that floor. As for residence halls, each floor having dormitories was assumed to have a coffee machine; the results are as follows: hall A (8), hall B (6), hall C (6), hall D (8), hall E (8), hall F (16), hall G (12), hall H (13), hall I (11), hall J (5), hall K (5), hall L (9), halls M and N (37), and hall O (8). The next area of interest was common dining areas; these areas were not dining halls because dining halls do not contain self-serve machines. Thus assumptions were disregarded for pay-per-meal dining halls and residence hall dining, which would contain floors already counted. The food facility (31) and food facility service (23) categories were the only areas considered due to the aforementioned reasons. The last potential spaces included meeting areas that were not occupying spaces already counted; the total count for this category was 47.

## Supplementary information

Supplementary Information.
