# Supplementary material for: Life Cycle Assessment of Compostable Coffee Pods: A US University Based Case Study
Source: Sci Rep. 2020 Jun 8;10:9158. doi: 10.1038/s41598-020-65058-1 (PMC7280196; doi:10.1038/s41598-020-65058-1)

Life Cycle Assessment of Compostable Coffee Pods: A US University Based Case Study

Komal Kooduvalli, Uday Vaidya, Soydan Ozcan

SUPPLEMENTARY MATERIAL

Supplementary Table S1. List of closest compostable pods to Knoxville

Company name	Location	Distance from Knoxville (mi)	Extra info: Organic (Y/N)	Cost: Highest count box & per pod*
Company A (Lowest price)	Lincoln, CA 95648	2439	Y & N	$55.99/160 pods => $0.35/pod
Company B (Closest distance)	Astoria, NY 11105	724	Y	32.99/40 pods --> $0.82/pod
Company C (Optimum distance and price)	Toronto, Ontario, Canada	733	Y	$9.99/20 (sale) -> 0.49/pod
Company D	Hayward, WI	964	Y	Case of 6 $36/72 pods => $0.5
Company E	Burnaby, British Columbia, Canada	2725	Y	55.99/72 pods --> $0.78/pod
Company F	Vancouver, British Columbia, Canada	2732	Y	7.99/12 pods --> $0.67/pod
Company G	Edmonds, WA 98020	2584	Y	$0.46/pod on Amazon
Company H	Kalaheo, HI 96741	4561	-	$8.48/12 --> $0.71/pod $43/72 -> 0.59/pod
Company I	Oceanside, NY 11572	736	-	$1375/2500 --> $0.55
Company J	Sherbrooke, QC J1H 5C5, Canada	1122	-	$0.58/pod on amazon
Company K	Miami, FL, 33133	880	-	$37.50/60 pods --> $0.625/pod
Company L	Newfoundland and Labrador, Canada	2380	-	$10.49/20 (Sale) -> $0.52/pod

**Prices are subject to vary. Bulk prices are assumed to be much smaller in comparison to the abovementioned retail prices*

The company that produces the conventional plastic coffee pod taken within the study is referred to as Company X

Keyword search terms: "compostable coffee pod"; "compostable pods"

Supplementary Table S2. Exported SimaPro results: CED single score based on weight of each pod component

SimaPro 9.0.0.33	Impact assessment	Date:	7/15/2019	Time:	10:44				
Project	Coffee Pods								
Calculation:	Compare								
Results:	Impact assessment								
Product 1:	0.2504 g Compostable lid (of project Coffee Pods)								
Product 2:	0.1290 g Compostable mesh (of project Coffee Pods)								
Product 3:	2.6828 g Compostable ring (of project Coffee Pods)								
Product 4:	0.2002 g Plastic filter (of project Coffee Pods)								
Product 5:	0.2462 g Plastic lid (of project Coffee Pods)								
Product 6:	2.5749 g Plastic shell (of project Coffee Pods)								
Method:	Cumulative Energy Demand V1.10 / Cumulative energy demand								
Indicator:	Single score								
Skip categories:	Never								
Default units:	Yes								
Exclude infrastructure processes:	No								
Exclude long-term emissions:	No								
Sorted on item:	Impact category								Highest value
Sort order:	Ascending								Lowest value
**Impact category**	**Unit**	**Compostable lid**	**Compostable mesh**	**Compostable ring**	**Plastic filter**	**Plastic lid**	**Plastic shell**	**EE of plastic pod**	**EE of compostable pod**
Total	MJ	2.43E-02	1.19E-02	2.97E-01	1.47E-02	3.30E-02	2.75E-01	3.23E-01	3.33E-01
Non renewable, fossil	MJ	1.40E-02	7.02E-03	1.88E-01	6.83E-03	2.86E-02	2.45E-01		
Non-renewable, nuclear	MJ	2.80E-03	1.31E-03	3.55E-02	8.54E-04	2.07E-03	2.53E-02		
Non-renewable, biomass	MJ	3.72E-07	5.01E-08	5.58E-07	5.94E-07	1.77E-04	1.50E-07		
Renewable, biomass	MJ	7.10E-03	3.37E-03	6.90E-02	6.77E-03	1.12E-03	2.75E-03		
Renewable, wind, solar, geothe	MJ	9.59E-05	4.48E-05	1.21E-03	4.21E-05	5.45E-05	4.87E-04		
Renewable, water	MJ	2.66E-04	1.24E-04	3.33E-03	2.09E-04	9.48E-04	2.10E-03		

Supplementary Table S3. Exported SimaPro results: TRACI characterization values based on weight of each pod component

SimaPro 9.0.0.33	Impact assessment	Date:	7/15/2019	Time:	11:00		
Project	Coffee Pods						
Calculation:	Compare						
Results:	Impact assessment						
Product 1:	0.2504 g Compostable lid (of project Coffee Pods)						
Product 2:	0.1290 g Compostable mesh (of project Coffee Pods)						
Product 3:	2.6828 g Compostable ring (of project Coffee Pods)						
Product 4:	0.2002 g Plastic filter (of project Coffee Pods)						
Product 5:	0.2462 g Plastic lid (of project Coffee Pods)						
Product 6:	2.5749 g Plastic shell (of project Coffee Pods)						
Method:	TRACI 2.1 V1.04 / US 2008						
Indicator:	Characterization						
Skip categories:	Never						
Exclude infrastructure processes:	No						
Exclude long-term emissions:	No						
Sorted on item:	Impact category						
Sort order:	Ascending						
Impact category	Unit	Compostable lid	Compostable mesh	Compostable ring	Plastic filter	Plastic lid	Plastic shell
Ozone depletion	kg CFC-11 eq	5.43E-11	2.64E-11	2.41E-09	3.18E-11	8.05E-11	1.91E-09
Global warming	kg CO2 eq	1.16E-03	5.80E-04	1.48E-02	3.84E-04	2.15E-03	9.70E-03
Smog	kg O3 eq	4.51E-05	2.12E-05	5.57E-04	2.54E-05	1.09E-04	4.24E-04
Acidification	kg SO2 eq	5.05E-06	2.45E-06	6.26E-05	1.92E-06	1.04E-05	3.63E-05
Eutrophication	kg N eq	5.54E-06	2.94E-06	6.68E-05	1.19E-06	4.26E-06	1.20E-05
Carcinogenics	CTUh	4.68E-11	2.25E-11	5.71E-10	2.04E-11	2.84E-10	3.27E-10
Non carcinogenics	CTUh	1.15E-10	4.92E-11	1.36E-09	8.57E-11	3.57E-10	7.07E-10
Respiratory effects	kg PM2.5 eq	3.27E-07	1.53E-07	3.90E-06	4.85E-07	1.52E-06	2.60E-06
Ecotoxicity	CTUe	5.11E-03	2.56E-03	6.03E-02	2.06E-03	1.59E-02	2.38E-02
Fossil fuel depletion	MJ surplus	1.44E-03	7.43E-04	1.99E-02	8.02E-04	2.60E-03	3.21E-02

Supplementary Table S4. Exported SimaPro results: CED single score based on current UT scenario

SimaPro 9.0.0.33	Impact assessment	Date:	Time:	11:14			
Project	Coffee Pods						
Calculation:	Analyze						
Results:	Impact assessment						
Product:	339896.25 g Cradle-to-grave plastic pods 1yr (UTK scenario) -Company X (of project Coffee Pods)						
Method:	Cumulative Energy Demand V1.10 / Cumulative energy demand						
Indicator:	Single score					*Condensed for graph as:*	
Skip categories:	Never					**Label**	
Default units:	Yes					Plastic components	36351
Exclude infrastructure processes:	No					Transportation (9.1 mi)	19
Exclude long-term emissions:	No					Landfill (59.4 mi)	418
Sorted on item:	Impact category					**Total**	**36788**
Sort order:	Ascending						
Impact category	Unit	Total	Plastic shell	Plastic lid	Plastic filter	Transport, lorry 7.5-16t, EURO5/US- US-EI U	Landfill scenario for pods
Total	MJ	3.68E+04	3.10E+04	3.71E+03	1.66E+03	1.90E+01	4.18E+02
Non renewable, fossil	MJ	3.21E+04	2.75E+04	3.22E+03	7.69E+02	1.81E+01	5.27E+02
Non-renewable, nuclear	MJ	3.08E+03	2.85E+03	2.33E+02	9.61E+01	7.37E-01	-1.00E+02
Non-renewable, biomass	MJ	2.01E+01	1.69E-02	2.00E+01	6.69E-02	4.55E-05	2.78E-03
Renewable, biomass	MJ	1.20E+03	3.10E+02	1.26E+02	7.62E+02	3.77E-02	-9.29E-01
Renewable, wind, solar, geothe	MJ	6.28E+01	5.48E+01	6.13E+00	4.74E+00	2.45E-02	-2.93E+00
Renewable, water	MJ	3.61E+02	2.36E+02	1.07E+02	2.35E+01	8.66E-02	-5.48E+00

Supplementary Table S5. Exported SimaPro results: TRACI characterization percentages based on current UT scenario. Percentages represent the fraction of the emissions generated from each component and life cycle stage relative to the total emissions from the system. The percentages in each category add up to 100%

Exported from SimaPro 9.0.0.33					
Exported on: 7/15/2019 at 11:39:08 AM					
Analyzing 3.4E5 g 'Plastic pods 1yr (UTK scenario) -Company X';					
Method: TRACI 2.1 V1.04 / US 2008 / Characterization					
Unit used: %					
**Label**	**Plastic shell**	**Plastic lid**	**Plastic filter**	**Transport, lorry 7.5-16t, EURO5/US- US-EI U**	**Landfill scenario for pods**
Ozone depletion	9.04E+01	3.81E+00	1.51E+00	9.75E-02	4.20E+00
Global warming	6.14E+01	1.36E+01	2.44E+00	6.81E-02	2.24E+01
Smog	6.58E+01	1.70E+01	3.94E+00	1.34E-01	1.31E+01
Acidification	7.09E+01	2.03E+01	3.76E+00	7.55E-02	4.98E+00
Eutrophication	3.19E+01	1.13E+01	3.16E+00	2.84E-02	5.36E+01
Carcinogenics	4.68E+01	4.06E+01	2.92E+00	7.40E-02	9.62E+00
Non carcinogenics	1.24E+01	6.26E+00	1.50E+00	2.75E-02	7.98E+01
Respiratory effects	5.27E+01	3.08E+01	9.83E+00	6.52E-02	6.54E+00
Ecotoxicity	6.25E+00	4.18E+00	5.42E-01	8.60E-03	8.90E+01
Fossil fuel depletion	8.83E+01	7.15E+00	2.21E+00	5.87E-02	2.33E+00

Supplementary Table S6. Exported SimaPro results: TRACI characterization values based on current UT scenario

Product:	339896.25 g Plastic pods 1yr (UTK scenario) -Company X (of project Coffee Pods)						
Method:	TRACI 2.1 V1.04 / US 2008						
Indicator:	Characterization						
Skip categories:	Never						
Exclude infrastructure processes:	No						
Exclude long-term emissions:	No						
Sorted on item:	Impact category						
Sort order:	Ascending						
Impact category	Unit	Total	Plastic shell	Plastic lid	Plastic filter	Transport, lorry 7.5-16t, EURO5/US- US-EI U	Landfill scenario for pods
Ozone depletion	kg CFC-11 eq	2.37E-04	2.15E-04	9.05E-06	3.58E-06	2.31E-07	9.98E-06
Global warming	kg CO2 eq	1.78E+03	1.09E+03	2.42E+02	4.33E+01	1.21E+00	3.99E+02
Smog	kg O3 eq	7.25E+01	4.77E+01	1.23E+01	2.86E+00	9.71E-02	9.53E+00
Acidification	kg SO2 eq	5.75E+00	4.08E+00	1.17E+00	2.16E-01	4.35E-03	2.86E-01
Eutrophication	kg N eq	4.25E+00	1.35E+00	4.79E-01	1.34E-01	1.20E-03	2.28E+00
Carcinogenics	CTUh	7.87E-05	3.68E-05	3.19E-05	2.30E-06	5.82E-08	7.57E-06
Non carcinogenics	CTUh	6.42E-04	7.95E-05	4.02E-05	9.64E-06	1.77E-07	5.12E-04
Respiratory effects	kg PM2.5 eq	5.55E-01	2.93E-01	1.71E-01	5.46E-02	3.62E-04	3.63E-02
Ecotoxicity	CTUe	4.28E+04	2.67E+03	1.79E+03	2.32E+02	3.68E+00	3.81E+04
Fossil fuel depletion	MJ surplus	4.09E+03	3.61E+03	2.92E+02	9.03E+01	2.40E+00	9.51E+01

Supplementary Table S7. Exported SimaPro results: CED single score based on transportation sensitivity for varying procurement distances. Company C is the suggested scenario with Company X being the currently used conventional pod scenario

SimaPro 9.0.0.33	Impact assessme	Date:	7/15/2019	Time:	12:01
Project	Coffee Pods				
Calculation:	Compare				
Results:	Impact assessment				
Product 1:	344497.5 g Compost scenario compostable pods only (UTK avoided product -1 yr) -Company C Scenario (of project Coffee Pods)				
Product 2:	344497.5 g Compost scenario compostable pods only (UTK avoided product -1 yr) 0 mi (of project Coffee Pods)				
Product 3:	344497.5 g Compost scenario compostable pods only (UTK avoided product -1 yr) 1000 mi (of project Coffee Pods)				
Product 4:	344497.5 g Compost scenario compostable pods only (UTK avoided product -1 yr) 250 mi (of project Coffee Pods)				
Product 5:	344497.5 g Compost scenario compostable pods only (UTK avoided product -1 yr) 500 mi (of project Coffee Pods)				
Product 6:	344497.5 g Landfill scenario compostable pods only (UTK -1yr) -Company C Scenario (of project Coffee Pods)				
Product 7:	344497.5 g Landfill scenario compostable pods only (UTK -1yr) 0 mi (of project Coffee Pods)				
Product 8:	344497.5 g Landfill scenario compostable pods only (UTK -1yr) 1000 mi (of project Coffee Pods)				
Product 9:	344497.5 g Landfill scenario compostable pods only (UTK -1yr) 250 mi (of project Coffee Pods)				
Product 10:	344497.5 g Landfill scenario compostable pods only (UTK -1yr) 500 mi (of project Coffee Pods)				
Product 11:	339896.25 g Plastic pods 1yr (UTK scenario) -Company X (of project Coffee Pods)				
Product 12:	339896.25 g Plastic pods 1yr (UTK scenario) 0 mi (of project Coffee Pods)				
Product 13:	339896.25 g Plastic pods 1yr (UTK scenario) 1000 mi (of project Coffee Pods)				
Product 14:	339896.25 g Plastic pods 1yr (UTK scenario) 250 mi (of project Coffee Pods)				
Product 15:	339896.25 g Plastic pods 1yr (UTK scenario) 500 mi (of project Coffee Pods)				
Method:	Cumulative Energy Demand V1.10 / Cumulative energy demand				
Indicator:	Single score				
Skip categories:	Never				
Default units:	Yes				
Exclude infrastructure processes:	No				
Exclude long-term emissions:	No				
Sorted on item:	Impact category				
Sort order:	Ascending				

Impact category	Unit	Compost scenario compostable pods only (UTK avoided product -1 yr) -Company C Scenario	Compost scenario compostable pods only (UTK avoided product - 1 yr) 0 mi	Compost scenario compostable pods only (UTK avoided product -1 yr) 1000 mi	Compost scenario compostable pods only (UTK avoided product -1 yr) 250 mi	Compost scenario compostable pods only (UTK avoided product -1 yr) 500 mi	Landfill scenario compostable pods only (UTK -1yr) - Company C Scenario	Landfill scenario compostable pods only (UTK -1yr) 0 mi	Landfill scenario compostable pods only (UTK -1yr) 1000 mi	Landfill scenario compostable pods only (UTK -1yr) 250 mi	Landfill scenario compostable pods only (UTK -1yr) 500 mi	Plastic pods 1yr (UTK scenario) - Company X	Plastic pods 1yr (UTK scenario) 0 mi	Plastic pods 1yr (UTK scenario) 1000 mi	Plastic pods 1yr (UTK scenario) 250 mi	Plastic pods 1yr (UTK scenario) 500 mi
Total	MJ	3.90E+04	3.75E+04	3.96E+04	3.80E+04	3.85E+04	3.95E+04	3.79E+04	4.00E+04	3.84E+04	3.90E+04	3.68E+04	3.68E+04	3.89E+04	3.73E+04	3.78E+04
Non renewable, fossil	MJ	2.50E+04	2.35E+04	2.55E+04	2.40E+04	2.45E+04	2.55E+04	2.41E+04	2.61E+04	2.46E+04	2.51E+04	3.21E+04	3.21E+04	3.40E+04	3.25E+04	3.30E+04
Non-renewable, nuclear	MJ	4.51E+03	4.45E+03	4.53E+03	4.47E+03	4.49E+03	4.42E+03	4.36E+03	4.44E+03	4.38E+03	4.40E+03	3.08E+03	3.07E+03	3.16E+03	3.09E+03	3.12E+03
Non-renewable, biomass	MJ	1.17E-01	1.13E-01	1.18E-01	1.14E-01	1.16E-01	1.17E-01	1.13E-01	1.18E-01	1.14E-01	1.16E-01	2.01E+01	2.01E+01	2.01E+01	2.01E+01	2.01E+01
Renewable, biomass	MJ	8.95E+03	8.94E+03	8.95E+03	8.94E+03	8.94E+03	8.94E+03	8.93E+03	8.94E+03	8.93E+03	8.94E+03	1.20E+03	1.20E+03	1.20E+03	1.20E+03	1.20E+03
Renewable, wind, solar, geothe	MJ	1.54E+02	1.52E+02	1.55E+02	1.53E+02	1.53E+02	1.51E+02	1.49E+02	1.51E+02	1.49E+02	1.50E+02	6.28E+01	6.28E+01	6.54E+01	6.34E+01	6.41E+01
Renewable, water	MJ	4.28E+02	4.20E+02	4.30E+02	4.23E+02	4.25E+02	4.20E+02	4.12E+02	4.22E+02	4.15E+02	4.17E+02	3.61E+02	3.61E+02	3.71E+02	3.63E+02	3.66E+02

Supplementary Table S8. Exported SimaPro results: TRACI characterization values based on transportation sensitivity for varying procurement distances

SimaPro 9.0.0.33	Impact assessment	Date:	7/15/2019	Time:	17:19
Project	Coffee Pods				
Calculation:	Compare				
Results:	Impact assessment				
Product 1:	344497.5 g Compost scenario compostable pods only (UTK avoided product -1 yr) -Company C Scenario (of project Coffee Pods)				
Product 2:	344497.5 g Compost scenario compostable pods only (UTK avoided product -1 yr) 0 mi (of project Coffee Pods)				
Product 3:	344497.5 g Compost scenario compostable pods only (UTK avoided product -1 yr) 1000 mi (of project Coffee Pods)				
Product 4:	344497.5 g Compost scenario compostable pods only (UTK avoided product -1 yr) 250 mi (of project Coffee Pods)				
Product 5:	344497.5 g Compost scenario compostable pods only (UTK avoided product -1 yr) 500 mi (of project Coffee Pods)				
Product 6:	344497.5 g Landfill scenario compostable pods only (UTK -1yr) -Company C Scenario (of project Coffee Pods)				
Product 7:	344497.5 g Landfill scenario compostable pods only (UTK -1yr) 0 mi (of project Coffee Pods)				
Product 8:	344497.5 g Landfill scenario compostable pods only (UTK -1yr) 1000 mi (of project Coffee Pods)				
Product 9:	344497.5 g Landfill scenario compostable pods only (UTK -1yr) 250 mi (of project Coffee Pods)				
Product 10:	344497.5 g Landfill scenario compostable pods only (UTK -1yr) 500 mi (of project Coffee Pods)				
Product 11:	339896.25 g Plastic pods 1yr (UTK scenario) -Company X (of project Coffee Pods)				
Product 12:	339896.25 g Plastic pods 1yr (UTK scenario) 0 mi (of project Coffee Pods)				
Product 13:	339896.25 g Plastic pods 1yr (UTK scenario) 250 mi (of project Coffee Pods)				
Product 14:	339896.25 g Plastic pods 1yr (UTK scenario) 500 mi (of project Coffee Pods)				
Product 15:	339896.25 g Plastic pods 1yr (UTK scenario) 1000 mi (of project Coffee Pods)				
Method:	TRACI 2.1 V1.04 / US 2008				
Indicator:	Characterization				
Skip categories:	Never				
Exclude infrastructure processes:	No				
Exclude long-term emissions:	No				
Sorted on item:	Impact category				
Sort order:	Ascending				

Impact category	Unit	Compost scenario compostable pods only (UTK avoided product -1 yr) - Company C Scenario	Compost scenario compostable pods only (UTK avoided product -1 yr) 0 mi	Compost scenario compostable pods only (UTK avoided product -1 yr) 1000 mi	Compost scenario compostable pods only (UTK avoided product -1 yr) 250 mi	Compost scenario compostable pods only (UTK avoided product -1 yr) 500 mi	Landfill scenario compostable pods only (UTK -1yr) - Company C Scenario	Landfill scenario compostable pods only (UTK -1yr) 0 mi	Landfill scenario compostable pods only (UTK -1yr) 1000 mi	Landfill scenario compostable pods only (UTK -1yr) 250 mi	Landfill scenario compostable pods only (UTK -1yr) 500 mi	Plastic pods 1yr (UTK scenario) - Company X	Plastic pods 1yr (UTK scenario) 0 mi	Plastic pods 1yr (UTK scenario) 250 mi	Plastic pods 1yr (UTK scenario) 500 mi	Plastic pods 1yr (UTK scenario) 1000 mi
Ozone depletion	kg CFC-11 eq	2.99E-04	2.80E-04	3.06E-04	2.87E-04	2.93E-04	3.09E-04	2.90E-04	3.16E-04	2.97E-04	3.03E-04	2.37E-04	2.37E-04	2.44E-04	2.50E-04	2.63E-04
Global warming	kg CO2 eq	1.87E+03	1.77E+03	1.90E+03	1.80E+03	1.84E+03	2.37E+03	2.27E+03	2.40E+03	2.30E+03	2.34E+03	1.78E+03	1.77E+03	1.81E+03	1.84E+03	1.91E+03
Smog	kg O3 eq	7.44E+01	6.64E+01	7.72E+01	6.91E+01	7.18E+01	8.77E+01	7.98E+01	9.06E+01	8.25E+01	8.52E+01	7.25E+01	7.24E+01	7.51E+01	7.78E+01	8.31E+01
Acidification	kg SO2 eq	7.95E+00	7.60E+00	8.08E+00	7.72E+00	7.84E+00	8.53E+00	8.17E+00	8.66E+00	8.29E+00	8.42E+00	5.75E+00	5.75E+00	5.87E+00	5.99E+00	6.23E+00
Eutrophication	kg N eq	8.55E+00	8.45E+00	8.58E+00	8.48E+00	8.51E+00	1.09E+01	1.08E+01	1.09E+01	1.08E+01	1.08E+01	4.25E+00	4.24E+00	4.28E+00	4.31E+00	4.38E+00
Carcinogenics	CTUh	7.67E-05	7.20E-05	7.85E-05	7.36E-05	7.52E-05	8.44E-05	7.97E-05	8.61E-05	8.13E-05	8.29E-05	7.87E-05	7.86E-05	8.02E-05	8.18E-05	8.50E-05
Non carcinogenics	CTUh	1.86E-04	1.71E-04	1.91E-04	1.76E-04	1.81E-04	7.05E-04	6.90E-04	7.10E-04	6.95E-04	7.00E-04	6.42E-04	6.42E-04	6.47E-04	6.51E-04	6.61E-04
Respiratory effects	kg PM2.5 eq	5.21E-01	4.91E-01	5.32E-01	5.01E-01	5.11E-01	5.59E-01	5.29E-01	5.69E-01	5.39E-01	5.49E-01	5.55E-01	5.55E-01	5.65E-01	5.75E-01	5.95E-01
Ecotoxicity	CTUe	7.99E+03	7.69E+03	8.10E+03	7.79E+03	7.90E+03	4.65E+04	4.62E+04	4.66E+04	4.63E+04	4.64E+04	4.28E+04	4.28E+04	4.29E+04	4.30E+04	4.32E+04
Fossil fuel depletion	MJ surplus	2.68E+03	2.48E+03	2.75E+03	2.55E+03	2.62E+03	2.77E+03	2.58E+03	2.85E+03	2.65E+03	2.71E+03	4.09E+03	4.08E+03	4.15E+03	4.22E+03	4.35E+03

Supplementary Table S9. Exported SimaPro results: CED single score based on EOL sensitivity with varying compost-to-landfill ratios

SimaPro 9.0.0.33	Impact assessment	Date:	7/15/2019	Time:	17:37			
Project	Coffee Pods							
Calculation:	Compare							
Results:	Impact assessment							
Product 1:	344497.5 g Compost scenario compostable pods only (UTK avoided product -1 yr) COMP100-LAND0 (of project Coffee Pods)							
Product 2:	344497.5 g Compost scenario compostable pods only (UTK avoided product -1 yr) COMP20-LAND80 (of project Coffee Pods)							
Product 3:	344497.5 g Compost scenario compostable pods only (UTK avoided product -1 yr) COMP50-LAND50 (of project Coffee Pods)							
Product 4:	344497.5 g Compost scenario compostable pods only (UTK avoided product -1 yr) COMP80-LAND20 (of project Coffee Pods)							
Product 5:	344497.5 g Landfill scenario compostable pods only (UTK -1yr) LAND100-COMP0 (of project Coffee Pods)							
Product 6:	339896.25 g Plastic pods 1yr (UTK scenario) -Company X (of project Coffee Pods)							
Product 7:	339896.25 g Plastic pods 1yr (UTK scenario) -TEST 733mi (of project Coffee Pods)							
Method:	Cumulative Energy Demand V1.10 / Cumulative energy demand							
Indicator:	Single score							
Skip categories:	Never							
Default units:	Yes							
Exclude infrastructure processes:	No							
Exclude long-term emissions:	No							
Sorted on item:	Impact category							
Sort order:	Ascending							
Impact category	Unit	Compost scenario compostable pods only (UTK avoided product -1 yr) COMP100-LAND0	Compost scenario compostable pods only (UTK avoided product -1 yr) COMP20-LAND80	Compost scenario compostable pods only (UTK avoided product -1 yr) COMP50-LAND50	Compost scenario compostable pods only (UTK avoided product -1 yr) COMP80-LAND20	Landfill scenario compostable pods only (UTK -1yr) LAND100-COMP0	Plastic pods 1yr (UTK scenario) -Company X	Plastic pods 1yr (UTK scenario) -TEST 733mi
Non renewable, fossil	MJ	2.50E+04	2.54E+04	2.53E+04	2.51E+04	2.55E+04	3.21E+04	3.35E+04
Non-renewable, nuclear	MJ	4.51E+03	4.44E+03	4.46E+03	4.49E+03	4.42E+03	3.08E+03	3.13E+03
Non-renewable, biomass	MJ	1.17E-01	1.17E-01	1.17E-01	1.17E-01	1.17E-01	2.01E+01	2.01E+01
Renewable, biomass	MJ	8.95E+03	8.94E+03	8.94E+03	8.94E+03	8.94E+03	1.20E+03	1.20E+03
Renewable, wind, solar, geothe	MJ	1.54E+02	1.51E+02	1.52E+02	1.53E+02	1.51E+02	6.28E+01	6.47E+01
Renewable, water	MJ	4.28E+02	4.21E+02	4.24E+02	4.26E+02	4.20E+02	3.61E+02	3.68E+02
Total	MJ	3.90E+04	3.94E+04	3.92E+04	3.91E+04	3.95E+04	3.68E+04	3.83E+04

Supplementary Table S10. Exported SimaPro results: TRACI characterization values based on EOL sensitivity with varying compost-to-landfill ratios

SimaPro 9.0.0.33	Impact assessment	Date:	7/15/2019	Time:	18:32			
Project	Coffee Pods							
Calculation:	Compare							
Results:	Impact assessment							
Product 1:	344497.5 g Compost scenario compostable pods only (UTK avoided product -1 yr) COMP100-LAND0 (of project Coffee Pods)							
Product 2:	344497.5 g Compost scenario compostable pods only (UTK avoided product -1 yr) COMP20-LAND80 (of project Coffee Pods)							
Product 3:	344497.5 g Compost scenario compostable pods only (UTK avoided product -1 yr) COMP50-LAND50 (of project Coffee Pods)							
Product 4:	344497.5 g Compost scenario compostable pods only (UTK avoided product -1 yr) COMP80-LAND20 (of project Coffee Pods)							
Product 5:	344497.5 g Landfill scenario compostable pods only (UTK -1yr) LAND100-COMP0 (of project Coffee Pods)							
Product 6:	339896.25 g Plastic pods 1yr (UTK scenario) -Company X (of project Coffee Pods)							
Product 7:	339896.25 g Plastic pods 1yr (UTK scenario) -TEST 733mi (of project Coffee Pods)							
Method:	TRACI 2.1 V1.04 / US 2008							
Indicator:	Characterization							
Skip categories:	Never							
Exclude infrastructure processes:	No							
Exclude long-term emissions:	No							
Sorted on item:	Impact category							
Sort order:	Ascending							
Impact category	Unit	Compost scenario compostable pods only (UTK avoided product -1 yr) COMP100-LAND0	Compost scenario compostable pods only (UTK avoided product -1 yr) COMP20-LAND80	Compost scenario compostable pods only (UTK avoided product -1 yr) COMP50-LAND50	Compost scenario compostable pods only (UTK avoided product -1 yr) COMP80-LAND20	Landfill scenario compostable pods only (UTK -1yr) LAND100-COMP0	Plastic pods 1yr (UTK scenario) -Company X	Plastic pods 1yr (UTK scenario) -TEST 733mi
Ozone depletion	kg CFC-11 eq	2.99E-04	3.07E-04	3.04E-04	3.01E-04	3.09E-04	2.37E-04	2.56E-04
Global warming	kg CO2 eq	1.87E+03	2.27E+03	2.12E+03	1.97E+03	2.37E+03	1.78E+03	1.87E+03
Smog	kg O3 eq	7.44E+01	8.50E+01	8.10E+01	7.70E+01	8.77E+01	7.25E+01	8.03E+01
Acidification	kg SO2 eq	7.95E+00	8.41E+00	8.24E+00	8.07E+00	8.53E+00	5.75E+00	6.10E+00
Eutrophication	kg N eq	8.55E+00	1.04E+01	9.71E+00	9.01E+00	1.09E+01	4.25E+00	4.34E+00
Carcinogenics	CTUh	7.67E-05	8.29E-05	8.06E-05	7.83E-05	8.44E-05	7.87E-05	8.33E-05
Non carcinogenics	CTUh	1.86E-04	6.01E-04	4.45E-04	2.89E-04	7.05E-04	6.42E-04	6.56E-04
Respiratory effects	kg PM2.5 eq	5.21E-01	5.51E-01	5.40E-01	5.28E-01	5.59E-01	5.55E-01	5.84E-01
Ecotoxicity	CTUe	7.99E+03	3.88E+04	2.73E+04	1.57E+04	4.65E+04	4.28E+04	4.31E+04
Fossil fuel depletion	MJ surplus	2.68E+03	2.76E+03	2.73E+03	2.70E+03	2.77E+03	4.09E+03	4.28E+03

Supplementary Table S11. Exported SimaPro results: CED single score values for projected wet weight scenarios with current and control distances

SimaPro 9.0.0.33	Impact assessment	Date:	7/15/2019	Time:	18:44		
Project	Coffee Pods						
Calculation:	Compare						
Results:	Impact assessment						
Product 1:	2655000 g Compost scenario compostable pods only (UTK avoided product -1 yr) -Company C Scenario + wet weight (of project Coffee Pods)						
Product 2:	2655000 g Compost scenario compostable pods only (UTK avoided product -1 yr) + wet weight + no distance (of project Coffee Pods)						
Product 3:	2655000 g Landfill scenario compostable pods only (UTK -1yr) -Company C Scenario + wet weight (of project Coffee Pods)						
Product 4:	2655000 g Landfill scenario compostable pods only (UTK -1yr) + wet weight + no distance (of project Coffee Pods)						
Product 5:	2171250 g Plastic pods 1yr (UTK scenario) -Company X + wet weight (of project Coffee Pods)						
Product 6:	2171250 g Plastic pods 1yr (UTK scenario) + wet weight + no distance (of project Coffee Pods)						
Method:	Cumulative Energy Demand V1.10 / Cumulative energy demand						
Indicator:	Single score						
Skip categories:	Never						
Default units:	Yes						
Exclude infrastructure processes:	No						
Exclude long-term emissions:	No						
Sorted on item:	Impact category						
Sort order:	Ascending						
Impact category	Unit	Compost scenario compostable pods only (UTK avoided product -1 yr) -Company C Scenario + wet weight	Compost scenario compostable pods only (UTK avoided product -1 yr) + wet weight + no distance	Landfill scenario compostable pods only (UTK -1yr) -Company C Scenario + wet weight	Landfill scenario compostable pods only (UTK -1yr) + wet weight + no distance	Plastic pods 1yr (UTK scenario) -Company X + wet weight	Plastic pods 1yr (UTK scenario) + wet weight + no distance
Total	MJ	1.82E+05	1.75E+05	1.86E+05	1.78E+05	1.35E+05	1.35E+05
Non renewable, fossil	MJ	1.17E+05	1.10E+05	1.21E+05	1.14E+05	1.18E+05	1.18E+05
Non-renewable, nuclear	MJ	2.10E+04	2.07E+04	2.03E+04	2.00E+04	1.09E+04	1.09E+04
Non-renewable, biomass	MJ	5.55E-01	5.37E-01	5.54E-01	5.37E-01	7.30E+01	7.30E+01
Renewable, biomass	MJ	4.18E+04	4.18E+04	4.17E+04	4.17E+04	4.36E+03	4.36E+03
Renewable, wind, solar, geothe	MJ	7.21E+02	7.12E+02	6.95E+02	6.86E+02	2.21E+02	2.20E+02
Renewable, water	MJ	2.00E+03	1.97E+03	1.94E+03	1.91E+03	1.30E+03	1.30E+03

Supplementary Table S12. Exported SimaPro results: TRACI characterization values for projected wet weight scenarios with current and control distances

SimaPro 9.0.0.33	Impact assessment	Date:	7/15/2019	Time:	18:51		
Project	Coffee Pods						
Calculation:	Compare						
Results:	Impact assessment						
Product 1:	2655000 g Compost scenario compostable pods only (UTK avoided product -1 yr) -Company C Scenario + wet weight (of project Coffee Pods)						
Product 2:	2655000 g Compost scenario compostable pods only (UTK avoided product -1 yr) + wet weight + no distance (of project Coffee Pods)						
Product 3:	2655000 g Landfill scenario compostable pods only (UTK -1yr) -Company C Scenario + wet weight (of project Coffee Pods)						
Product 4:	2655000 g Landfill scenario compostable pods only (UTK -1yr) + wet weight + no distance (of project Coffee Pods)						
Product 5:	2171250 g Plastic pods 1yr (UTK scenario) -Company X + wet weight (of project Coffee Pods)						
Product 6:	2171250 g Plastic pods 1yr (UTK scenario) + wet weight + no distance (of project Coffee Pods)						
Method:	TRACI 2.1 V1.04 / US 2008						
Indicator:	Characterization						
Skip categories:	Never						
Exclude infrastructure processes:	No						
Exclude long-term emissions:	No						
Sorted on item:	Impact category						
Sort order:	Ascending						
Impact category	Unit	Compost scenario compostable pods only (UTK avoided product -1 yr) -Company C Scenario + wet weight	Compost scenario compostable pods only (UTK avoided product -1 yr) + wet weight + no distance	Landfill scenario compostable pods only (UTK -1yr) -Company C Scenario + wet weight	Landfill scenario compostable pods only (UTK -1yr) + wet weight + no distance	Plastic pods 1yr (UTK scenario) -Company X + wet weight	Plastic pods 1yr (UTK scenario) + wet weight + no distance
Ozone depletion	kg CFC-11 eq	1.40E-03	1.31E-03	1.48E-03	1.39E-03	8.92E-04	8.91E-04
Global warming	kg CO2 eq	8.43E+03	7.97E+03	1.23E+04	1.18E+04	7.56E+03	7.56E+03
Smog	kg O3 eq	3.36E+02	2.99E+02	4.39E+02	4.02E+02	2.90E+02	2.90E+02
Acidification	kg SO2 eq	3.63E+01	3.46E+01	4.07E+01	3.91E+01	2.17E+01	2.17E+01
Eutrophication	kg N eq	3.99E+01	3.94E+01	5.78E+01	5.73E+01	2.17E+01	2.17E+01
Carcinogenics	CTUh	3.58E-04	3.36E-04	4.17E-04	3.95E-04	3.07E-04	3.07E-04
Non carcinogenics	CTUh	8.67E-04	8.00E-04	4.87E-03	4.80E-03	3.74E-03	3.74E-03
Respiratory effects	kg PM2.5 eq	2.43E+00	2.29E+00	2.72E+00	2.58E+00	2.12E+00	2.12E+00
Ecotoxicity	CTUe	3.74E+04	3.60E+04	3.34E+05	3.33E+05	2.60E+05	2.60E+05
Fossil fuel depletion	MJ surplus	1.25E+04	1.16E+04	1.33E+04	1.23E+04	1.51E+04	1.51E+04

Supplementary Table S13. Exported SimaPro results: CED single score values for projected wet weight scenarios with equal pod weights

SimaPro 9.0.0.33	Impact assessment	Date:	7/15/2019	Time:	18:56
Project	Coffee Pods				
Calculation:	Compare				
Results:	Impact assessment				
Product 1:	1000 kg Compost scenario compostable pods only (UTK avoided product -1 yr) 0 mi (of project Coffee Pods)				
Product 2:	1000 kg Landfill scenario compostable pods only (UTK -1yr) 0 mi (of project Coffee Pods)				
Product 3:	1000 kg Plastic pods 1yr (UTK scenario) 0 mi (of project Coffee Pods)				
Method:	Cumulative Energy Demand V1.10 / Cumulative energy demand				
Indicator:	Single score				
Skip categories:	Never				
Default units:	Yes				
Exclude infrastructure processes:	No				
Exclude long-term emissions:	No				
Sorted on item:	Impact category				
Sort order:	Ascending				
Impact category	Unit	Compost scenario compostable pods only (UTK avoided product -1 yr) 0 mi	Landfill scenario compostable pods only (UTK -1yr) 0 mi	Plastic pods 1yr (UTK scenario) 0 mi	
Total	MJ	1.09E+05	1.10E+05	1.08E+05	
Non renewable, fossil	MJ	6.83E+04	6.98E+04	9.43E+04	
Non-renewable, nuclear	MJ	1.29E+04	1.26E+04	9.05E+03	
Non-renewable, biomass	MJ	3.28E-01	3.28E-01	5.90E+01	
Renewable, biomass	MJ	2.60E+04	2.59E+04	3.52E+03	
Renewable, wind, solar, geothe	MJ	4.42E+02	4.32E+02	1.85E+02	
Renewable, water	MJ	1.22E+03	1.20E+03	1.06E+03	

Supplementary Table S14. Exported SimaPro results: TRACI characterization values for projected wet weight scenarios with equal pod weights

SimaPro 9.0.0.33	Impact assessment	Date:	7/15/2019	Time:	19:01
Calculation:	Compare				
Results:	Impact assessment				
Product 1:	1000 kg Compost scenario compostable pods only (UTK avoided product -1 yr) 0 mi (of project Coffee Pods)				
Product 2:	1000 kg Landfill scenario compostable pods only (UTK -1yr) 0 mi (of project Coffee Pods)				
Product 3:	1000 kg Plastic pods 1yr (UTK scenario) 0 mi (of project Coffee Pods)				
Method:	TRACI 2.1 V1.04 / US 2008				
Indicator:	Characterization				
Skip categories:	Never				
Exclude infrastructure processes:	No				
Exclude long-term emissions:	No				
Sorted on item:	Impact category				
Sort order:	Ascending				
Impact category	Unit	Compost scenario compostable pods only (UTK avoided product - 1 yr) 0 mi	Landfill scenario compostable pods only (UTK -1yr) 0 mi	Plastic pods 1yr (UTK scenario) 0 mi	
Ozone depletion	kg CFC-11 eq	0.000814046	0.000843162	0.000698025	
Global warming	kg CO2 eq	5132.867159	6584.606788	5221.395414	
Smog	kg O3 eq	192.8340476	231.5189433	213.096171	
Acidification	kg SO2 eq	22.05605736	23.7265217	16.91557486	
Eutrophication	kg N eq	24.52209892	31.27357885	12.48694541	
Carcinogenics	CTUh	0.000208961	0.000231209	0.00023123	
Non carcinogenics	CTUh	0.000496983	0.002004091	0.001887993	
Respiratory effects	kg PM2.5 eq	1.426059648	1.535728832	1.632303392	
Ecotoxicity	CTUe	22326.36492	134207.2085	125817.2254	
Fossil fuel depletion	MJ surplus	7204.34308	7485.751116	12018.19984	

Supplementary Table S15. Coffee pod numbers derived from floor plan estimations (UT facilities portal)

	Floor Number											
**Offices**	**1**	**2**	**3**	**4**	**5**	**6**	**7**	**8**	**B**	**G**	**P1**	**P2**
**Faculty**	11	15	15	17	9	6				2		
**Library Faculty**	3	5	3	2	1	1				1		
**Staff**	15	18	17	11	8	5	3	1	1	4	1	1
**Clerical**	45	52	34	31	18	13	4	4	5	6		1
**Visitor**	36	33	18	12	2	5						
**TOTAL**	495											

**Residential**	**Hall A**	**Hall B**	**Hall C**	**Hall D**	**Hall E**	**Hall F**	**Hall G**	**Hall H**	**Hall I**	**Hall J**	**Hall K**	**Hall L**	**Halls M & N**	**Hall O**
**Floors to fit assump.**	8	6	6	8	8	16	12	13	11	5	5	9	37	8
**TOTAL**	152													

**Food Areas**	**Assum. #**
**Food Facility**	31
**Food Facility Service**	23
**TOTAL**	54
**Running Total**	748

**Meeting**	**1**	**2**	**3**	**4**	**5**	**6**	**7**	**8**	**Deductions**
**Floor cnt.**	27	21	10	5	2	4	2	1	25
**TOTAL**	47								

*Deducted areas outside of UTK's immediate campus and overlapping talleys for libraries and residential areas.

Example screenshot of portal

Code changes per building. Respective floors in each building are grouped

Floor number satisfies the assumption. Only one assumed per floor as per manuscript expectations.

Identifiers under Name category. These will show residence hall names and college groups.
Keywords to block assumptions:
-Dining Hall
-(Any group without a name)
-Area outside of study (UT Chattanooga, Martin, etc.)

Rooms for: 01.300-10.1

Building Code	*Floor Code*	*Room Number*	*Room Area ft²*	*Room Standard*	*College ID*	*Name*	*Fund ID*	*Name*	*Prorate*
50110100	02	211	206.46		70110	College of Arts and Sciences	E011068	Psychology	NONE
50110100	02	215A	116.47		70110	College of Arts and Sciences	E011068	Psychology	NONE
50110100	02	215B	107.72		70110	College of Arts and Sciences	E011068	Psychology	NONE
50110100	02	215C	107.04		70110	College of Arts and Sciences	E011068	Psychology	NONE
50110100	02	215E	105.97		70110	College of Arts and Sciences	E011068	Psychology	NONE
50110100	02	215F	107.09		70110	College of Arts and Sciences	E011068	Psychology	NONE
50110100	02	215G	108.45		70110	College of Arts and Sciences	E011068	Psychology	NONE
50110100	02	221E	117.44		70110	College of Arts and Sciences	E011068	Psychology	NONE
50110100	02	223	184.26		70110	College of Arts and Sciences	E011068	Psychology	NONE
50110100	02	225A	149.17		70110	College of Arts and Sciences	E011068	Psychology	NONE
50110100	03	301A	128.42		70110	College of Arts and Sciences	E011068	Psychology	NONE
50110100	03	301B	138.84		70110	College of Arts and Sciences	E011068	Psychology	NONE
50110100	03	301C	126.75		70110	College of Arts and Sciences	E011068	Psychology	NONE
50110100	03	301D	125.19		70110	College of Arts and Sciences	E011068	Psychology	NONE
50110100	03	301E	140.83		70110	College of Arts and Sciences	E011068	Psychology	NONE
50110100	03	301F	115.74		70110	College of Arts and Sciences	E011068	Psychology	NONE
50110100	03	301G	116.05		70110	College of Arts and Sciences	E011068	Psychology	NONE
50110100	03	301H	107.32		70110	College of Arts and Sciences	E011068	Psychology	NONE
50110100	03	301I	125.10		70110	College of Arts and Sciences	E011068	Psychology	NONE
50110100	03	303A	115.57		70110	College of Arts and Sciences	E011068	Psychology	NONE
50110100	03	303B	116.18		70110	College of Arts and Sciences	E011068	Psychology	NONE
50110100	03	303C	114.93		70110	College of Arts and Sciences	E011068	Psychology	NONE
50110100	03	303D	148.39		70110	College of Arts and Sciences	E011068	Psychology	NONE
50110100	03	303E	132.41		70110	College of Arts and Sciences	E011068	Psychology	NONE
50110100	03	303F	130.72		70110	College of Arts and Sciences	E011068	Psychology	NONE
50110100	03	303G	143.11		70110	College of Arts and Sciences	E011068	Psychology	NONE
50110100	03	303H	134.70		70110	College of Arts and Sciences	E011068	Psychology	NONE
50110100	03	307E	160.82		70110	College of Arts and Sciences	E011068	Psychology	NONE

Supplementary Table S16. Annual waste allocation data from the UT Sustainability Office

Waste Allocation FY2018 UTK Data			
Information received from Recycling Manager on March 5, 2019			
Waste	*Amount (tons)*	*Percentage*	
Regular waste going to landfill -minus construction and demolition	6426.79	67	
Amount diverted from landfill	3175.82	33	
**Total waste generated on campus**	**9602.61**	**100**	
Amount composted in FY2018 from food waste (food, compost)	447.34		
Amount composted in FY2018 from green waste (leaves, manure, wood chips, brush)	729.9		
**Total**	**1177.24**	**12**	This would be a part of the 33% mentioned above
Total amount composted in FY2018	1177.24	12	
Amount not composted	8425.37	88	100
Total amount diverted from landfill	3175.82	33	
Total amount of waste landfilled	6426.79	67	100

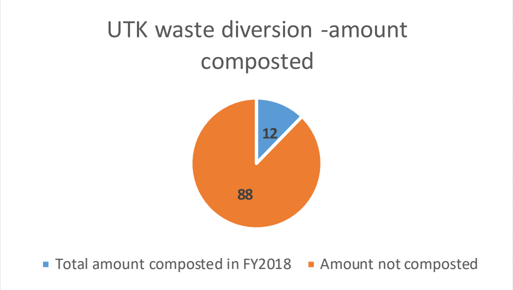

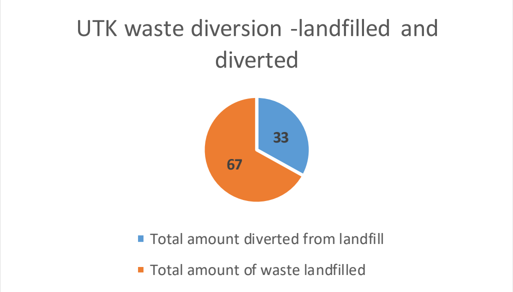

Supplement: Supplementary file 1 — Supplementary Information. [file 41598_2020_65058_MOESM1_ESM.pdf]
